# Inhibitory Learning with Bidirectional Outcomes: Prevention Learning or Causal Learning in the Opposite Direction?

**DOI:** 10.5334/joc.266

**Published:** 2023-03-10

**Authors:** Julie Y. L. Chow, Jessica C. Lee, Peter F. Lovibond

**Affiliations:** 1 UNSW, Sydney, Australia; 2 The University of Sydney, Australia

**Keywords:** causal learning, prevention, causal structure, feature negative, bidirectional outcomes

## Abstract

Influential models of causal learning assume that learning about generative and preventive relationships are symmetrical to each other. That is, a preventive cue directly prevents an outcome from occurring (i.e., “direct” prevention) in the same way a generative cue directly causes an outcome to occur. However, previous studies from our lab have shown that many participants do not infer a direct prevention causal structure after feature-negative discrimination (A+/AB–) with a unidirectional outcome (Lee & Lovibond, 2021). Melchers et al. (2006) suggested that the use of a bidirectional outcome that can either increase or decrease from baseline, encourages direct prevention learning. Here we test an alternative possibility that a bidirectional outcome encourages encoding of a *generative* relationship in the *opposite* direction, where B directly causes a decrease in the outcome. Thus, previous evidence of direct prevention learning using bidirectional outcomes may instead be explained by some participants inferring an “Opposite Causal” structure. In two experiments, participants did indeed report an opposite causal structure. In Experiment 1, these participants showed the lowest outcome predictions when B was combined with a novel cause in a summation test, and lowest outcome predictions when B was presented alone. In Experiment 2, B successfully blocked learning to a novel cue that was directly paired with a reduction in the outcome, and this effect was strongest among participants who endorsed an Opposite Causal structure. We conclude that previous evidence of direct prevention learning using bidirectional outcomes may be a product of excitatory rather than inhibitory learning.

## Introduction

The ability to learn about causal or predictive relationships between events in the environment is important for adaptation. It allows the person or animal to anticipate significant outcomes like the presence of threat or rewards. In a generative causal relationship, the presence of the candidate cause (sometimes referred to as a cue) directly predicts the presence of the target outcome. For example, the presence of rain clouds signals incoming rain. Also important, however, is the ability for people to predict the *absence* of significant outcomes. Preventive relationships involve the absence of the outcome when the cue is present.

Although there are many ways in which people might acquire causal beliefs, for example through verbal instruction, we are specifically interested in how people learn from covariation between events in the environment — that is, evidence collected from their own experience with the cue and the outcome. Learning from covariation has a longstanding history in associative learning and cognitive psychology. The basic assumption here is that causation can be inferred from statistical patterns such as the co-occurrence or non-occurrence of events (e.g., Cheng, 1997; Shanks, 2004). When human participants are exposed to contingencies where the probability of the outcome occurring increases or decreases in the presence of the cue, they reliably rate the cue as generating or preventing the outcome respectively (Shanks & Dickinson, 1988). Accordingly, most theories of associative learning or causal inference assume that learning about a preventive relationship is largely the same as learning about a generative causal relationship, that is, causation and prevention are equivalent but opposite in sign (e.g., Rescorla-Wagner (RW) model, Rescorla & Wagner, 1972; Power PC Theory, Cheng, 1997, Novick & Cheng, 2004; Causal Support, Griffiths & Tenenbaum, 2005).

However, the idea that causation and prevention are equivalent but opposite in sign disguises an important asymmetry in the conditions required to both learn and express these two types of relationship. Generative causal learning can be achieved by pairing a cue with an outcome, but preventive learning cannot be achieved by simply pairing a cue with the absence of an outcome. This is because the latter situation is indeterminate – the cue could be associated with the absence of an indefinite number of different outcomes. Instead, preventive learning requires some expectation that a particular outcome should occur, either through another causal agent or the context (i.e. base rate). In the laboratory, prevention learning is often studied using a feature negative (FN) design, in which a causal cue A is followed by the outcome except on trials where a preventive feature B is also present (A+/AB– discrimination).

Although associative and cognitive models differ in the assumed psychological processes underlying learning, implicit in both associative and cognitive models of learning is that the conditions or experimental procedure for acquiring preventive relationships are not symmetrical to causal relationships. For example, in the Rescorla-Wagner model, inhibitory learning is driven by expectation of an outcome that does not occur (negative prediction error). The asymmetry is also reflected in the use of different equations for generative and preventive learning in Cheng’s (1997) Power PC theory. Cheng addresses this asymmetry explicitly and asserts that “treating a preventive cause as the mirror image of a generative cause…is problematic” (1997, p.388). To cite an example provided by Cheng, if a researcher is interested in the efficacy of a treatment in *causing* headaches, they need only administer the treatment to a population to determine whether it is effective or not. In contrast, the efficacy of a treatment in *preventing* headaches cannot be determined by presenting the treatment if the patient taking the treatment does not already have a headache (i.e., causal agent is missing). Under these conditions, the preventive power of the cue cannot be determined. Similarly, generative causal learning can be expressed behaviourally (for example in anticipatory responding) simply by presenting the cue, but preventive learning is typically behaviourally silent (Zimmer-Hart & Rescorla, 1974; Konorski, 1948), and additionally requires the presence of a causal cue in order to be detected – in the associative literature this is known as a summation test (Pavlov, 1927).

Despite this asymmetry in how we learn about generative vs preventive relationships, both cognitive and associative models appear to assume that once learnt, the representations underlying generative and preventive learning are essentially the same, and differ only in the direction of their action on the outcome. Cognitive models like Power PC (Cheng, 1997), and Bayesian models that propose similar causal power principles (e.g., Griffiths & Tenenbaum, 2005), typically imply that generative and preventive causes are both encoded by the learner as acting directly on the outcome. In line with this interpretation, when both generative and preventive links are included in the same causal diagram, they are both depicted by a direct link between a cue and an outcome but with a positive or negative weight to indicate causation or prevention (e.g., Carroll et al., 2013; Gong & Bramley, 2021).

Certain models are even more explicit in their assumption that preventive and generative learning differ only in the direction of their action. In this paper, we will focus on the Rescorla-Wagner model (Rescorla & Wagner, 1972). The RW model has been widely influential in human and animal associative learning and makes clear, testable predictions. This model assumes that a person or non-human animal tracks the covariation between events through mental links connecting representations of these events in memory. The strength of the mental links between cue and outcome change flexibly according to experience. A cue that reliably predicts the presence of an outcome (i.e., an excitor) comes to directly activate the representation of that outcome, acquiring positive associative strength. In contrast, a cue that predicts the omission of an outcome (an inhibitor) is said to directly suppress the outcome representation, acquiring negative associative strength (Konorski, 1948; Rescorla, 1969). The RW model is clear in presenting these two types of learning as opposite to each other. Excitation and inhibition are seen as lying on a single dimension of associative strength, with excitors having positive associative strength and inhibitors having negative associative strength. Researchers have previously noted the similarities between factors that influence Pavlovian conditioning in animals and human contingency learning (e.g., Alloy & Abramson, 1979; Shanks & Dickinson, 1988), suggesting that these associative processes, i.e., ability for a cue to activate or suppress mental representation of an outcome, may govern the extent to which we judge a putative cause and effect to be causally related (Hume, 1888; Dickinson, 1980). Thus, although originally developed to account for animal conditioning data, associative learning models like the RW model have been suggested to provide a good account of causal learning in humans (Dickinson, Shanks & Evenden, 1984).

We have previously argued that the RW model maps onto the idea of prevention learning in so far as excitors and inhibitors act directly on the same outcome representation; that is, causation and prevention are symmetric opposites of each other both in terms of the continuum of associative strength and the structure underlying learning (Lee & Lovibond, 2021). Our interpretation of the RW model is consistent with other contemporary associative accounts of inhibition, including those made by Rescorla and Wagner separately (e.g., Rescorla, 1969; Brandon, Vogel & Wagner, 2003). We refer to this causal structure as “direct prevention”. Note that we will refrain from using the term “conditioned inhibition” since this term is often used to describe a learning phenomenon (i.e., a negative feature passing a summation test) that is consistent with multiple underlying causal structures (Lee & Lovibond, 2021; see Sosa, 2022 for a review). However, an alternative causal structure for inhibitory learning has also been identified in the associative literature by Rescorla (1985) and Holland & Lamarre (1984). These researchers showed that in an A+/AB– FN discrimination, a learner may encode B not as directly inhibiting the outcome but as modulating or gating the relationship between A and the outcome. That is, A alone causes the outcome to occur, but when B is also present, A no longer causes the outcome to occur. This is known as negative occasion setting in associative learning (Bonardi, Robinson & Jennings, 2017; Fraser & Holland, 2019). We will refer to this causal structure as “modulatory”, since it explicitly describes the action of a preventive cue as modulating the action of a causal cue rather than acting directly on the outcome.

Evidence for modulatory learning comes from studies showing that the ability of the feature to modulate another cue’s relationship with the outcome is largely independent of its own relationship with the outcome (Fraser & Holland, 2019). Thus, modulatory learning provides a formal alternative causal structure to direct prevention learning that not only specifies the need for a generative cause to be present during learning, but also assumes this cause is explicitly incorporated into the learned causal structure.

**Figure 1:**
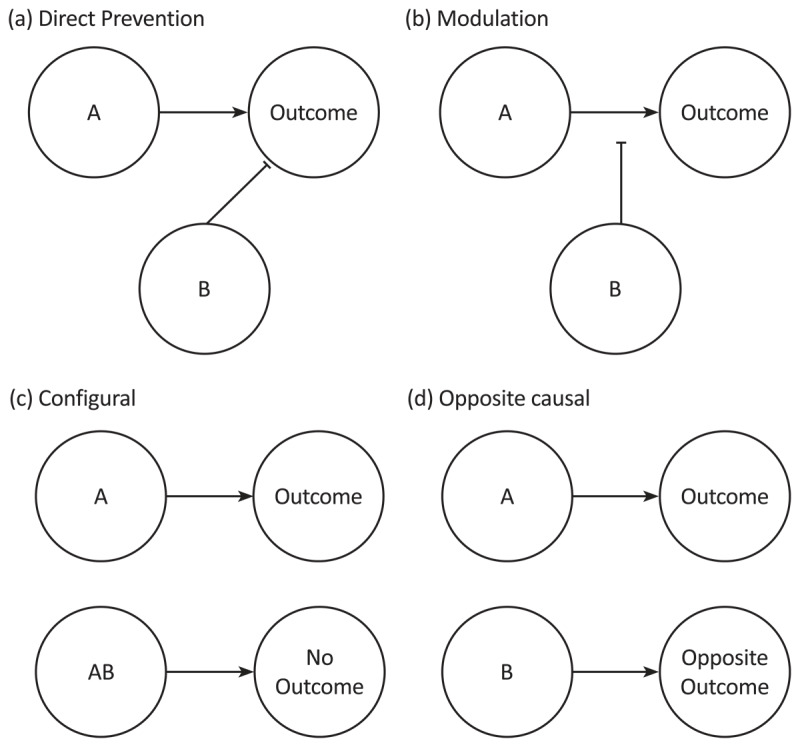
Diagrammatic Representation of All Four Causal Structures. *Note*: Pointed arrowheads represent an excitatory connection, and flat arrowheads represent an inhibitory connection.

To our knowledge, direct prevention and modulation are the two most clearly specified causal structures that represent a negative relationship between a cue and an outcome. When cues are presented in an A+/AB– FN arrangement, the causal scenario is ambiguous and it is unclear which causal structure is “correct”. In the animal conditioning literature, a key determinant of what is learned is whether the AB compound is presented serially or simultaneously. Animals typically show modulatory learning (negative occasion setting) to the feature when the compound stimuli are presented serially (A is followed by the outcome unless preceded by cue B, i.e., A+/B –> A–), and something like direct prevention learning (conditioned inhibition) when the stimuli are presented simultaneously (A+/AB–; e.g., Holland & Gory, 1986). However, there is relatively little evidence for direct prevention learning in humans. In our work on self-reported causal structures after FN training, we have observed substantial individual differences in the content of inhibitory learning (Lee & Lovibond, 2021). We have previously shown that in addition to a direct prevention causal structure, many participants report learning a modulatory causal structure regardless of whether a serial or simultaneous design is used (Lee & Lovibond, 2021; Lovibond & Lee, 2021). A third group of participants reported learning only about which outcome will occur with each combination of stimuli; we classify this group of participants as Configural learners, in the sense that they learn about all cues presented together (e.g., in a compound) rather than trying to infer their individual roles (see Figure 1). Importantly, suppression of outcome predictions in a summation test, where B is presented with a different causal cue, is usually incomplete in human causal learning (Karazinov & Boakes, 2007; Lee & Livesey, 2012), and much smaller in magnitude compared to non-human animals. According to the RW model, if B has acquired negative associative strength after FN discrimination, one would expect the summation of opposing associative strengths (positive associative strength of the causal cue and negative associative strength of B) to produce much stronger suppression of outcome predictions than is typically observed. Further evidence against the idea of arithmetic summation of opposing causal strengths comes from recent work demonstrating that summation is heavily influenced by the similarity between trained and transfer stimuli, suggesting that the summation test is better characterised as a test of generalisation (Chow et al., 2022). Finally, in both humans and animals, repeated presentations of a preventive cue alone do not lead to a loss of its preventive properties, in contradiction to the predictions of the RW model (Lovibond, Chow, Tobler & Lee, 2022; Zimmer-Hart & Rescorla, 1974). Thus, evidence of direct prevention learning is limited, especially in human causal learning (and maybe also in animals).

Melchers, Wolff and Lachnit (2006) proposed that the difficulty in demonstrating direct prevention learning of the type proposed in the RW model in humans could be a product of the type of outcome used in causal learning studies. These researchers pointed out that most studies employ outcomes that can only vary unidirectionally (e.g. an allergic reaction that either occurs or does not occur, but there is no anti-allergic reaction). They suggested that unidimensional outcomes do not accurately reflect the symmetrical continuum of associative strengths described in the RW model. Instead, they proposed employing bidirectional outcomes in causal learning tasks, where the outcome could increase *or* decrease relative to some baseline. According to Melchers et al. (2006), this procedure would allow the feature B to elicit an expectation that the outcome would *decrease* below baseline, an outcome they described as being in line with the assumptions of the RW model (i.e., negative associative strength). Thus, they argued that if a bidirectional outcome were employed during a FN discrimination, participants would be more likely to infer that B directly prevents the outcome from occurring. They provided some evidence for this claim by showing that the inhibitory properties of the feature B could be extinguished by presenting the feature alone (B–) when the outcome was bidirectional but not when it was unidirectional. The idea here is that when a bidirectional outcome is used, negative associative links could increase from a negative value to zero, allowing the feature to lose its inhibitory power.

However, there is an alternative way in which people could interpret the role of the feature when FN training (A+/AB–) is carried out with a bidirectional outcome. If we consider the outcome below baseline as a distinct outcome in itself, it is plausible that instead of acquiring prevention learning, where the feature B prevents the outcome signalled by A, participants might instead infer that B directly causes the *opposite* outcome to that elicited by A. For example, if cue A predicted an increase in outcome level and AB predicted no change, participants might infer that B directly caused a *decrease* in the outcome. This “opposite causal” structure is distinct from both of the inhibitory structures, prevention and modulation, described earlier. Opposite causal learning is different from direct prevention learning since it involves a *generative* causal link between the cue and the opposite outcome (in associative terms, the feature activates a representation of the opposite outcome, rather than suppressing a representation of the actual outcome presented). An opposite causal structure is also distinct from a modulatory causal structure since it involves a *direct* link between the feature and the outcome, and therefore the feature is able to be active on its own without an existing source of excitation (e.g., another causal agent present) for its effect to be seen. In summary, the opposite causal structure is distinct from genuine inhibitory structures like direct prevention and modulation, as it involves a *direct excitatory* connection between the feature and an (opposite) outcome. In contrast, both direct prevention and modulation involve the inhibition or suppression of an outcome, either directly (direct prevention) or by gating the relationship between a causal cue and an outcome (modulation). Diagrammatic representations of all four causal structures potentially inferred in a FN discrimination task (direct prevention, modulation, configural and opposite causal) are shown in Figure 1.

The goal of the present study was to test this alternative explanation that when presented with bidirectional outcomes in a FN discrimination, some participants acquire opposite excitatory learning to the feature. Across two experiments, we tested this hypothesis by incorporating the new opposite causal structure into our previous method for assessing individual differences in causal structure inference (see Experiment 1 Procedure). We used a modified allergist task with the same causal scenario used by Melchers et al. (2006) and Lotz and Lachnit (2009), with hormone levels as outcomes that either increased, decreased or were unchanged. Our primary hypothesis was that a FN discrimination with a bidirectional outcome could be solved by assuming that the feature directly causes a reduction in hormone level (opposite causal) rather than preventing the hormone level from increasing. Thus, we expected a proportion of participants to not only report such an opposite causal structure to the feature alone (i.e., that it *causes a decrease* in hormone level), but also that these individuals would show the greatest transfer effect in a summation test compared to participants who reported an inhibitory (direct prevention or modulation) causal structure, where as noted earlier transfer is often incomplete. These findings would suggest that we have identified a novel causal structure when a bidirectional outcome is used, and demonstrate that opposite causal is distinct from other known inhibitory structures.

## Experiment 1

The aim of Experiment 1 was to explore whether participants endorse an “opposite causal” structure to explain the role of the feature B in an A+/AB– feature negative discrimination when the outcome is bidirectional in nature. In our design and the design of Melchers et al (2006) and Lotz & Lachnit (2009), the term “bidirectional” is used to refer to outcomes that can increase or decrease from baseline, while “unidirectional” refers to (binary) outcomes that can only increase from baseline (outcome absent/present). In their design, Melchers et al. (2006) included an individual cue F that was followed by no change (F^0^) in their unidirectional outcome group but by a reduction in hormone level (F–) in their bidirectional outcome group. Another aim of our study was to test whether giving such direct experience with a reduction in hormone level was critical to establish the bidirectional nature of the outcome, or whether simply instructing participants that it could change in either direction would be sufficient. Therefore, we included a similar cue I in our training phase, which we labelled a “reference” cue, and followed it by no change in hormone level in one group (the No Reference Group) and by a reduction in hormone level in a second group (Reference group). In our experiment, however, *both* groups were instructed that the outcome was bidirectional and could increase, stay the same or decrease after a meal.

Apart from the reference cue I, participants were exposed to a range of food cues A–H that followed the same design as in our previous experiments on prevention learning (e.g., Lee & Lovibond, 2021) and implemented an A+/AB– feature negative discrimination in addition to training a control cue D and filler cues (see Table 1). These cues were all followed by a hormone increase or no change. After the training phase, participants were asked to make predictions about novel compounds in which a separately trained excitor C was combined with the feature B, as well as with a control cue D (summation test). This comparison assessed the transfer of B’s properties from the training excitor A to the test excitor C, relative to the control cue D. We hypothesised that participants in the Reference group would show a larger difference in CB vs CD ratings, as well as more negative prediction ratings to B alone, compared to participants who only ever saw an increase in hormone levels.

**Table 1:** Procedure of Experiment 1.


TRAINING	TEST PREDICTIONS	CAUSAL RATINGS	OPEN-ENDED QUESTION	FORCED-CHOICE CAUSAL STRUCTURE ASSESSMENT

A+ AB^0^	A AB B	A B	B	B
C+	C CB CD CI	C		
DE^0^	DE D E	D E		
F^0^ GH+	F	F		
I^0^/I–	I J	I J		


*Note*: + = an increase in hormone level, 0 = no change, and – = a decrease.

Our previous work on feature negative learning in causal judgment with unidirectional outcomes suggested that humans infer *different* causal structures about the feature B in a FN discrimination, regardless of whether AB was presented simultaneously or serially. Some participants reported that B directly prevents the outcome (Direct Prevention), consistent with the RW conception of prevention learning, whereas others reported the role of B as determining whether the causal cue A will cause the outcome to occur (Modulation), much like the modulatory role of a negative occasion setter (Lee & Lovibond, 2021). A third group of participants reported learning only about which outcome will occur with each combination of stimuli (Configural). Importantly, we found corresponding differences in transfer of B’s inhibitory properties to the test excitor C, where participants in the Direct Prevention subgroup provided the greatest transfer (lowest prediction ratings to the CB compound compared to control compound CD), Configural participants showed the least amount of transfer, and Modulation participants were in between the two (see also Glautier & Brudan, 2019). In the present experiment, where the outcome was bidirectional, we predicted that some participants would infer a fourth causal structure, namely an Opposite Causal structure. In order to differentiate between empirical and theoretical aspects in this paper, we will use capital letters when referring to empirical data involving subgroup categories (e.g., Opposite Causal) and lowercase letters for the abstract theoretical causal structures.

In this study, we predicted that not only would there be more participants in the Reference group who reported an Opposite Causal structure compared to the No Reference group, but this subgroup of participants would also show strongest transfer compared to all other subgroups, indexed by greater difference in ratings for CB relative to CD. Our rationale here was that Opposite Causal participants would be more confident in predicting transfer since they construed the feature as directly causing the opposite effect to the test excitor, where the similarity of A and C should not matter.

The Reference group experienced cue I paired with a decrease in hormone level (I–), whereas the No Reference group experienced cue I paired with no change in hormone level (I^0^). Column headings describe each phase of the experiment in sequence from left to right, beginning with the Training phase and ending with the Forced-choice causal structure assessment. All stimuli presented in each phase (and their associated outcomes) are denoted below the relevant column heading.

### Method

#### Participants

One-hundred and eighty nine participants recruited through Prolific (64 female, *M_age_* = 30.3, *SD* = 10.4) participated in this study in exchange for monetary payment (20min at £6GBP/hr).

#### Apparatus & Stimuli

The experiment was programmed using the jsPsych library (de Leeuw, 2015). Cue stimuli, which included the image of the food and a text description, were presented on a blank background that was 300 pixels wide by 300 pixels high. A total of 10 experimental cues were used in this study (cues A–J); the assignment of cues was randomised across participants. Outcome stimuli consisted of a verbal description of the change in hormone level (e.g. hormone level: no change) in bold text. Outcome stimuli were presented on a blank background 609 pixels wide and 300 pixels high.

#### Procedure

The task procedure reported here followed previous work from our lab (Lee & Lovibond, 2021; Chow, Lee & Lovibond, 2022), apart from the bidirectional nature of the outcome and the prediction scale.

##### Training phase

Participants were asked to imagine they were a doctor investigating which foods were causing fluctuations in hormone level in “Mr X”. Participants were told that excessive levels of the hormone are linked to a particular disease, and changes in hormone level are thought to be related to diet. On each trial, participants were shown different meals that Mr X had eaten, and asked to predict whether Mr X’s hormone level would increase, decrease, or experience no change after eating that meal. Predictions were made on a scale from “Definitely DECREASE” to “Definitely INCREASE”, with a midpoint of “No Change”. Ratings were recorded on a bidirectional numerical scale from –100 to +100.

Once participants had made a prediction, the “Continue” button appeared at the bottom of the scale and they were able to continue to the next screen. The prediction scale was then replaced with outcome feedback in text (hormone level increase, decrease or no change), which remained on screen for 2 seconds before a 2-second intertrial interval where all cues disappeared from the screen.

Training trials consisted of 3 blocks with 2 presentations of each trial type in each block (see Table 1). The order of presentation for compound cues was counterbalanced within-block (e.g. AB and BA), such that participants saw both orders of presentation in a randomised order (AB first or BA first). Trial types were also randomised in a way that participants never saw two identical trials presented successively. In total, participants completed 42 training trials with 6 presentations of each trial type.

##### Outcome prediction at test

After completing the training phase, participants were asked to continue making predictions about changes in Mr X’s hormone level, but were told they would no longer be presented with feedback. The prediction scale used in this phase was identical to that presented during training. Participants were shown summation compounds consisting of the separately trained excitor C with the trained inhibitor B (CB), control cue D (CD), and reference cue I (CI), as well as familiar cues from the previous phase (A–I) and a novel cue J. Each trial type was presented twice, and the left-to-right order of presentation for compounds cues was again counterbalanced.

##### Causal ratings

Following the test predictions, participants were given instructions about the causal judgement phase of the study. They were asked to rate the extent to which they thought different foods caused or prevented an increase in hormone level in Mr X. Participants were informed that the rating scale in this phase was different to the prediction scale they had seen in previous phases. The causal rating scale ranged from “Strongly PREVENTED an increase” to “Strongly “CAUSED an increase” with a midpoint of “No effect”. These ratings were recorded on a bidirectional numerical scale from –100 to +100. The wording of the causal rating question was chosen to match that used in our previous studies on feature negative learning with unidirectional outcomes (e.g., Lee & Lovibond, 2021), and also so that it would be consistent with any of the hypothesised causal structures.

##### Open ended question

After completing the causal judgement phase, participants were asked a single open-ended question about the critical cue B. Participants were shown an image of cue B at the top of the screen, followed by a text field box where they could explain what they had learned about the role of cue B. Participants were encouraged not to leave the field box empty.

##### Causal structure assessment

In the final phase of the study, participants were asked to assess the role of cue B in a 4-alternative forced-choice (4AFC) question. An image of cue B was presented at the top of the screen, and participants were asked to select the option that best described what they thought about the role of cue B. Three of the options were similar to those used in Lee & Lovibond (2021), Lovibond & Lee (2021) and Chow, Lee & Lovibond (2022). Specifically, the Modulation option was “It prevented an increase in hormone level caused by specific foods”, the Direct Prevention option was “It prevented an increase in hormone level in general”, and the Configural option was “It is hard to know the exact role of individuals foods such as this one. I concentrated on remembering which combinations of foods caused changes in hormone level and worked from there”. In addition, we included a fourth option to capture an opposite causal structure, where cue B is thought to produce the outcome in the opposite direction (i.e. hormone decrease). The phrasing of the Opposite Causal option was “It caused a decrease in hormone level”. The order of presentation of the four options was randomised between participants. After making a selection, participants could proceed to the final screen where they were asked if they had written anything down during the course of the experiment. Participants were encouraged to provide an honest response and were told that their response would not affect their eligibility to be paid.

#### Statistical analysis

The primary measure of interest was the outcome prediction ratings for summation compounds CB and CD. To test these differences, and in particular whether they were systematic differences as a function of group, we analysed the data with three orthogonal contrasts using the *afex* (Singmann et al., 2018) and *emmeans* (Lenth, 2019) packages in R. We compared 1) average ratings for compound CB compared to CD, 2) average ratings for participants in the Reference group compared to the No Reference group for compounds CB and CD, and 3) the interaction between these two contrasts. We also included simple effects to test the CB vs CD difference for the Reference group and the No Reference group separately. We also directly compared average ratings for the feature B for participants in the No Reference vs Reference group. For analysis of causal ratings, we compared ratings for cues B vs D (averaged over group), ratings for Reference vs No Reference group (averaged over cues B and D), and the interaction between the two contrasts.

Data from the training phase were analysed with planned contrasts that tested 1) ratings for cues followed by hormone increase to those followed by no change common to both groups (common predictive vs non-predictive cues), 2) linear trend over the 6 presentations, 3) average ratings for Reference vs No Reference group, and 4) all interactions between these contrasts. Contrasts 2–4 were repeated for the comparison between reference cue I and common non-predictive cues.

For all the contrasts described above, we also included a between-subject factor of inferred causal structure determined by participants response on the 4-AFC question. We tested two planned contrasts: 1) Opposite Causal compared to the average of all other subgroups, and 2) Configural subgroup compared to the average of the two inhibitory subgroups (Direct Prevention and Modulation). All contrasts were tested as main effects (averaged over all other factors), as well as in interaction with all contrasts on other factors. These contrasts were selected to compare the subgroup of participants who endorsed an Opposite Causal structure to all other subgroups, as this structure is novel to the present study. We have also previously found the Configural subgroup to produce qualitatively different pattern of results compared to Modulation and Prevention subgroups (Lee & Lovibond, 2021); this is tested in the second contrast. These participants tend to report only remembering the outcomes of the stimulus combinations and remain agnostic about the effects of B alone. The phrasing of the Configural option in the 4-AFC question also differed from the other options in that it did not involve making any inferences about the causal status of B, and was therefore the more conservative of the options. Thus, of the four causal structures assessed in this study, we predicted the strongest transfer in the Opposite Causal subgroup, and the weakest transfer in the Configural subgroup, with Modulation and Prevention subgroups in between. We did not include contrasts comparing the two inhibitory subgroups in the present study as we have previously argued that differences between participants who report a Direct Prevention and Modulation causal structure does not reflect qualitative differences in what is inferred, but rather quantitative differences in their willingness to generalise properties of the feature B to a novel test excitor (Chow, Lee & Lovibond, 2022).

Finally, we also included a chi-square test of independence to compare participants’ causal structure selection as a function of group to determine whether direct experience with a negative outcome influenced the proportion of participants who inferred an opposite excitatory causal structure to B.

The full dataset for the two experiments reported here is publicly available at the Open Science Framework, and can be accessed at https://osf.io/9at4b/.

### Results

To recap our specific hypotheses for this experiment, we predicted that more participants would report an Opposite Causal structure in the Reference group, where they were explicitly presented with a cue that led to hormone level decrease, compared to the No Reference group. We additionally predicted that Opposite Causal participants would show greatest transfer in a summation test, indexed by greater difference in prediction ratings for CB compared to CD, as well as more negative prediction ratings to B alone, compared to all other subgroups. For brevity, figures presented on all test measures only include the primary cues of interest. For participants’ ratings on all stimuli presented at test, see online Supplemental Materials.

#### Exclusion criteria

As in our previous published studies (Lee & Lovibond, 2021; Lovibond & Lee, 2021; Chow, Lee & Lovibond, 2022), participants’ data were excluded from analysis if they reported writing down information during the task or if they failed to meet the training criterion. To pass the training criterion, all participants were required to provide 1) average rating > 75 for positive predictive cues and, 2) average rating between –25 and 25 for non-predictive cues. Participants in the Reference group were additionally required to provide an average rating < –75 for the reference cue I (cue I was a non-predictive cue in the No Reference group). In addition to the two criteria described above, participants were also presented with an instruction check prior to starting the task (see Supplemental Materials for the exact wording). Failure to provide a correct response on all three questions resulted in the program restarting at the first instruction screen. Participants who failed this instruction check more than twice (instructions were repeated at least three times) were excluded from analysis. Of the 189 participants recruited, 17 participants admitted to writing something down during the study, an additional 11 further participants failed the instruction check, and an additional 20 failed to meet the training criterion. After applying all three exclusions, 141 eligible datasets remained; 71 datasets from participants in the No Reference group, and 70 datasets from participants in the Reference group.

#### Causal structure assessment

For consistency with previous published studies from our lab, we defined subgroups based on participants’ forced choice causal structure selection and not their open-ended responses. A breakdown of subgroup categorisation for participants in the No Reference and Reference groups is shown in Table 2. Although there were some differences between the groups in the pattern of causal structure choices, a chi-squared test of independence showed no statistical difference in reported causal structure as a function of group, c^2^ (3) = 6.37, *p* = .095. Thus, contrary to our hypothesis, we found no significant difference in the proportion of participants reporting an Opposite Causal structure as a function of group.

**Table 2:** Number of participants in each group as a function of their 4-AFC selection.


GROUP	CAUSAL STRUCTURE	NUMBER OF PARTICIPANTS

No Reference	Configural	20

	Modulation	23

	Opposite Causal	19

	Prevention	9

Reference	Configural	28

	Modulation	19

	Opposite Causal	9

	Prevention	14


#### Training

**Figure 2:**
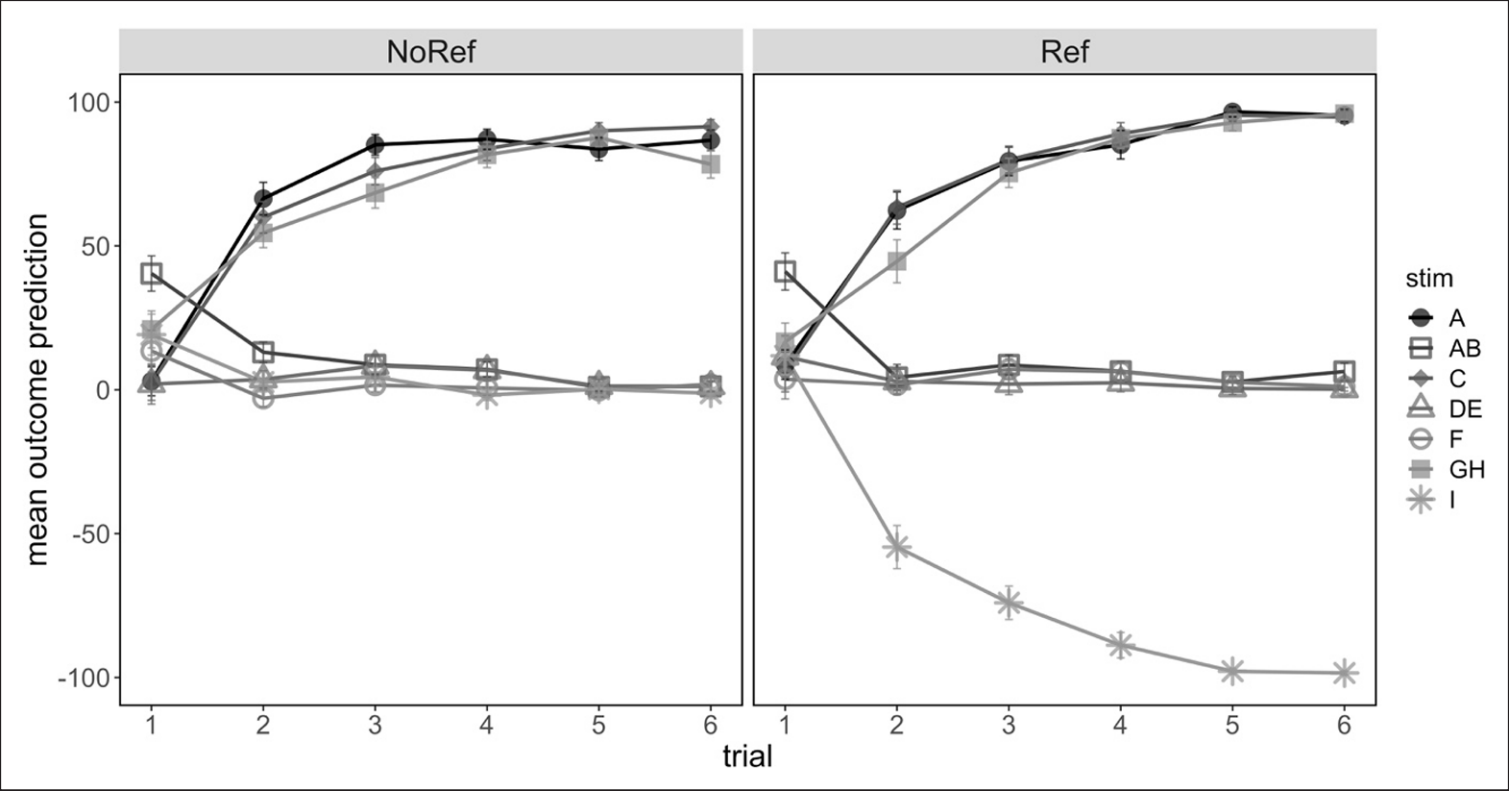
Mean Outcome Prediction (±SE) During Training For Participants In No Reference And Reference Group In Experiment 1. *Note*: Filled symbols denote stimuli that predicted hormone level increase, and unfilled symbols denote stimuli that predicted no change (or hormone level decrease in the case of cue I in the Reference condition).

Figure 2 shows participants’ average predictions across the six presentations of each trial type, separated by group. Analysis of training predictions showed a significant overall effect of cue type comparing common predictive to non-predictive cues, *F*(1,133) = 1487, *p* < .001, η_p_^2^ = .918, that also interacted with linear trend of presentation, *F*(1,133) = 423.3, *p* < .001, η_p_^2^ = .761. These findings suggest that participants successfully learned over trials which cues were predictive of an increase in hormone level, and which cues produced no change. Importantly there was no significant interaction with group, *F*(1,133) = 1.46, *p* = .228, η_p_^2^ = .011; learning for the common predictive and non-predictive cues progressed similarly for the two groups. Comparison of ratings for the reference cue I compared to the non-predictive cues showed a main effect of cue type, *F*(1,133) = 405.7, *p* < .001, η_p_^2^ = .753, which interacted significantly with linear trend of presentation, *F*(1,133) = 77.8, *p* < .001, η_p_^2^ = .369. Importantly we also found a three-way interaction between cue type, linear trend and group, *F*(1,133) = 65.3, *p* < .001, η_p_^2^ = .329, confirming that learning about the reference cue progressed differently for the two groups, with participants in the Reference group successfully learning across successive trials that cue I predicted a decrease in hormone level.

Causal structure comparisons in the Training phase revealed only a marginally significant interaction that reflected slightly stronger discrimination between the common predictive and non-predictive cues in the Opposite Causal subgroup compared to the other three subgroups, *F*(1,133) = 5.03, *p* = .027, η_p_^2^ = .036. No other main effects or interactions were significant, *F*s < 1. Training data broken down by causal structure subgroup can be found in Supplemental Materials.

#### Outcome prediction at test

**Figure 3:**
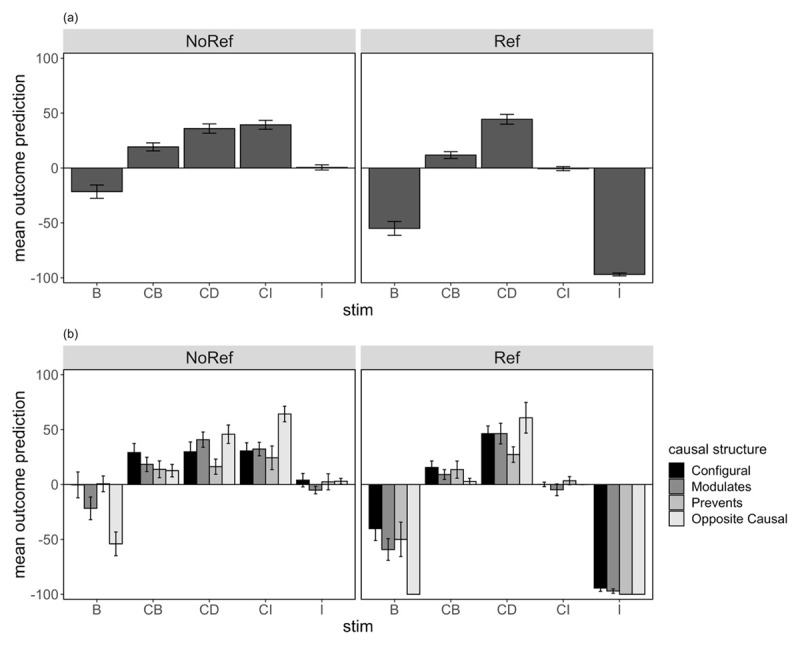
Average Outcome Prediction at Test (±SE) for Critical Summation Compounds **(a)** as a Group Average, and **(b)** Separated by Causal Structure Subgroup, for the No Reference and Reference Group Respectively in Experiment 1.

Each participant’s test predictions were averaged across the two presentations for each trial type. Figure 3 shows the mean outcome predictions for primary stimuli of interest, B, CB, CD, CI, and I, separated by group (3a) and broken down by causal structure subgroups (3b). We were primarily interested in participants’ ratings for summation compounds CB and CD, as well as test predictions for B alone. Comparison of participants’ ratings to cue I alone were also included as a manipulation check.

Contrasts comparing CB and CD ratings showed a main effect of cue, *F*(1,133) = 43.2, *p* < .001, η_p_^2^ = .245, which also interacted significantly with group, *F*(1,133) = 7.17, *p* = .008, η_p_^2^ = .051. These results indicate that overall, participants showed successful transfer of B’s properties from A to C, and the CB–CD difference was greater for participants who saw a reference cue leading to hormone decrease than for those who never saw hormone decrease as an outcome. There was no main effect of group, *F* < 1.

Importantly, we found the CB–CD difference to interact with causal structure, in particular when comparing the Opposite Causal subgroup to all other subgroups, *F*(1,133) = 8.68, *p* = .004, η_p_^2^ = .061. As hypothesised, the Opposite Causal subgroup showed more suppressed ratings to CB compared to CD relative to the other subgroups. Importantly, there were no further interaction with group as a factor, suggesting that individual differences in transfer as a function of inferred causal structure were insensitive to the reference cue manipulation. No other contrasts were significant, largest *F*(1,133) = 2.03, *p* = .157, η_p_^2^ = .015 (see Supplemental Materials for additional results involving subgroup contrasts).

A similar comparison of ratings to B alone at test showed a main effect of group, *F*(1,133) = 24.1, *p* < .001, η_p_^2^ = .154, with lower predictive ratings in the Reference group than No Reference group. There was also a causal structure difference when we compared the Opposite Causal subgroup to all other subgroups, *F*(1,133) = 19.4, *p* < .001, η_p_^2^ = .127. No other contrasts or interaction involving causal structure subgroup was significant, *Fs* < 1. Together, these results show that participants who saw a separate cue that caused a decrease in hormone level were more likely to predict that B alone would result in hormone decrease than participants who never experienced hormone decrease as an outcome. Furthermore, as we hypothesised, participants who inferred an Opposite Causal structure were also more likely to predict hormone level decrease in the presence of B alone, and showed greater transfer of B’s properties in a summation test. Critically, however, the effect of direct experience with a negative outcome and causal structure were additive, as we found no interaction between causal structure and group.

Unsurprisingly, analysis of ratings to cue I alone showed a main effect of group, *F*(1,133) = 1144.7, *p* < .001, η_p_^2^ = .898, with lower ratings in the Reference group than the No Reference group. No other contrasts tested were significant, largest *F*(1,133) = 2.27, *p* = .135, η_p_^2^ = .017.

#### Causal ratings

**Figure 4:**
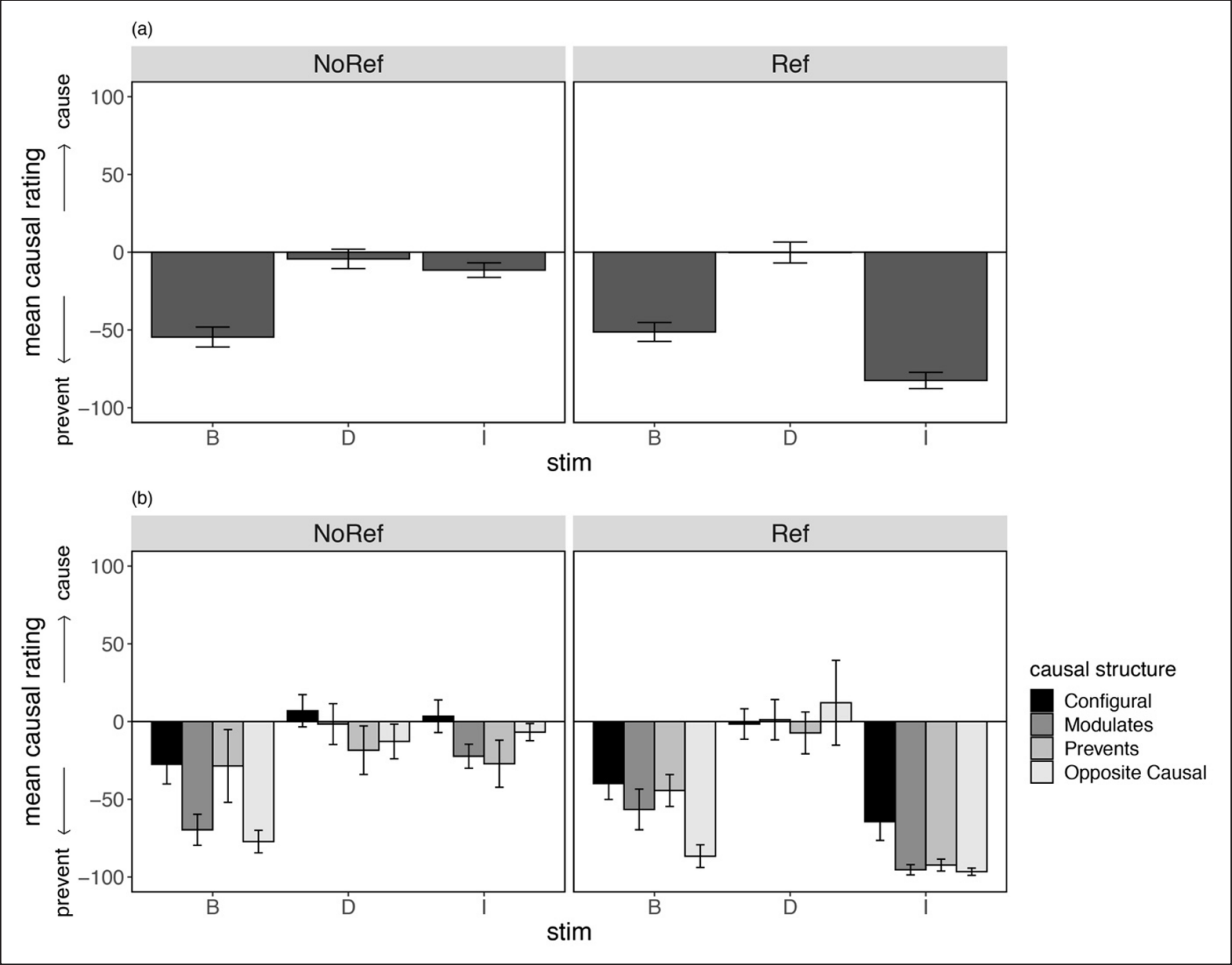
Mean Causal Ratings at Test (±SE) for Critical Stimuli Only for Participants in the No Reference and Reference Group as a **(a)** Group Mean, and **(b)** Separated By Causal Structure in Experiment 1. *Note*: Causal ratings were made on cause-prevent scale, from –100 (Strongly prevented an increase) to +100 (Strongly caused an increase) with a midpoint of 0 (No effect).

Figure 4 illustrates causal ratings for cues B, D and I, for each of the groups separately (4a) and separated by causal structure subgroup (4b). This test differed from the outcome prediction test in that participants were asked to rate the ability for each individual cue to cause or prevent the outcome from occurring.

Comparison of mean ratings for cues B and D revealed a main effect of cue, *F*(1,133) = 51.7, *p* < .001, η_p_^2^ = .280, with more negative (preventive) ratings for B relative to D averaged across groups. There was no main effect of group, and no interaction between group and cue, *F*s < 1. Contrasts comparing the different causal structure subgroups revealed a small but significant difference in mean ratings for the Opposite Causal subgroup relative to all other subgroups (averaged over cues B and D), *F*(1,133) = 4.72, *p* = .032, η_p_^2^ = .034, and an interaction between this comparison and cue type (B vs D), *F*(1,133) = 5.27, *p* = .023, η_p_^2^ = .038. No other causal structure interactions were significant, largest *F*(1,133) = .923, *p* = .657, η_p_^2^ = .001. Together these results suggest some differences as a function of inferred causal structure on ratings of B’s ability to prevent the outcome—the difference in causal ratings to B compared to D was significantly greater for Opposite Causal compared to all others subgroups. These findings did not appear to differ statistically as a function of group.

Analysis of participants’ ratings for cue I revealed lower (more preventive) ratings in the Reference group compared to the No reference group, *F*(1,133) = 102.9, *p* < .001, η_p_^2^ = .436, and higher ratings for the configural subgroup compared to the average of the two inhibitory subgroups, *F*(1,133) = 13.2, *p* < .001, η_p_^2^ = .090. No other comparisons were statistically significant, *Fs* < 1.

### Discussion

Experiment 1 showed that when the feature B in an A+/AB– FN discrimination was presented in compound with the predictive cue C at test, ratings were lower compared to when C was paired with a control cue, D. This difference in summation test ratings was greater when participants had direct experience with a separate cue that led to a decrease in hormone level (Reference group), compared to those who had never seen a negative outcome (No Reference group). Participants in the Reference group were also more likely to predict a decrease in hormone level when cue B was presented alone compared to participants in the No Reference group. However, we did not find the same difference between groups on causal ratings for cue B relative to cue D. We also did not find a difference in the proportion of participants reporting each causal structure as a function of group. Thus, although experience with hormone level decrease produced stronger transfer effects in the summation test, direct experience with a negative outcome was not necessary for participants to infer an opposite excitatory causal structure for B. A possible reason for this result is that simply presenting a bidirectional prediction scale was sufficient to encourage some participants to consider that certain foods might lead to a decrease in hormone level, even if they had no direct evidence of this in the experiment. This suggests that the inclination for some participants to infer an opposite causal structure in a FN discrimination might be greater than previously expected.

Contrasts comparing the different causal structure subgroups also showed differences in summation test ratings as a function of self-reported causal structure for cue B. As predicted, we found greater transfer for participants who endorsed an Opposite Causal structure compared to all other subgroups in the summation test. Participants who reported an Opposite Causal structure also provided more negative prediction ratings to cue B compared to all other subgroups, suggesting that participants who inferred an opposite generative relationship between B and the outcome were more likely to predict a decrease in hormone levels in the presence of B alone, and to show greater transfer of B’s properties to a novel causal cue. Together these results show that when the outcome has the potential to be negative, some participants infer an opposite causal relationship between the negative feature and the outcome, and this type of learning is distinct from prevention and modulatory learning.

In summary, in addition to the causal structures we have investigated in our previous work, Experiment 1 provided initial evidence for a possible fourth causal structure inferred by participants in a FN discrimination when the outcome is bidirectional. Importantly, these results are the first to show that strong transfer in a summation test and negative prediction ratings to the feature alone when a bidirectional outcome is used are largely driven by participants who inferred an opposite excitatory causal structure to B (B causes a decrease in hormone levels) rather than direct prevention learning as previously proposed (B prevents hormone levels from increasing). The possibility of a negative outcome seems to allow participants to infer an opposite excitatory causal structure for B whereby it is seen as directly causing a decrease in outcome level.

## Experiment 2

If our conception of an opposite causal structure is correct, then participants who endorse this structure should treat the feature as equivalent to a cue that has been explicitly trained to predict a reduction in the outcome. In Experiment 2, we tested this proposition by examining the degree to which the feature would block learning about a novel cue that was directly paired with a decrease in hormone level. The blocking procedure is commonly used in the associative learning literature to study the effects of cue competition (Kamin, 1969). The idea here is that humans and animals judge the causal relationship between two events by considering other potential causal cues in the environment, and all cues present concurrently compete for predictive value. When an outcome is already well predicted by a cue, for example if participants already expect hormone levels to decrease in the presence of B, learning about the predictive significance of another cue present at the same time is impaired.

In this study, the feature was first presented in a FN discrimination like in Experiment 1. Then, in a Blocking phase, the feature B was presented in compound with a novel cue (X) and the compound was followed by a decrease in hormone level (BX–). We hypothesised that participants who had inferred an opposite causal structure for B would show greater blocking of X compared to a control cue Y that was also novel in phase 2 and followed by the same decrease in hormone level but whose partner cue was never part of a FN discrimination (DY–). This is because these participants should learn that B alone will cause a hormone level decrease, which should effectively compete with the novel cue X for association with the observed decrease in hormone level. In other words, when BX is presented with hormone level decrease, the outcome is not surprising and learning about X is blocked. In contrast, the genuinely inhibitory structures (direct prevention, modulation) would not be expected to support strong blocking. In the case of modulation, participants are thought to learn that the feature B modulates the relationship between the training excitor A and an increase in hormone level, which is not directly relevant to the Blocking manipulation. Indeed, a modulator should make no assumption about the ability for B to be active by itself without an excitor present. In the case of direct prevention, participants are thought to learn that the feature prevents the outcome produced by the training excitor A (hormone level increase), therefore a preventer might learn that B alone would lead to no change in hormone levels, since B prevents hormone levels from increasing. When presented with the BX compound for the first time, the outcome (hormone level decrease) should be surprising since it is not predicted by the presence of B or X, and the cues compete equally for association. Thus, we expected to find greater evidence of blocking to X in the Opposite Causal subgroup relative to all other subgroups.

The design of Experiment 2 is shown in Table 3. Participants were presented with a similar predictive learning task to Experiment 1, with foods as cues and changes in hormone level as the outcome. The hormone level could again increase, decrease or stay the same on each trial. However, unlike in Experiment 1, hormone level changes were presented as numeric values. A feature negative contingency was set up such that cue A reliably predicted an increase in hormone level by 20 units (A+20), and simultaneous presentation of cues A and B resulted in no change in hormone level (AB0). To enhance transfer of inhibition, all participants were presented with a reference cue that predicted a decrease in hormone level by 20 units (C–20). This reference cue was also used in a comparison to B at test in order to determine if participants treated B in the same way as a cue that was directly paired with a reduction in hormone level, which we predict might be the case for participants who endorse an Opposite Causal structure. DE was included to provide a control stimulus for feature B, where cue D was similarly presented in compound with another cue and that the compound was always followed by no change in hormone level (DE0). Cue F was included as a filler cue (F0) to prevent participants from learning that all single cues led to some change in hormone level. A critical difference in the training phase of Experiment 2 compared to Experiment 1 was the inclusion of an additivity design, where cues G and H individually predicted an increase in hormone level by 20 units (G+20, H+20), and GH in compound predicted an even larger increase in hormone level of 40 units (GH+40). The inclusion of a magnitude additivity manipulation has previously been shown to enhance blocking (Lovibond, Been, Mitchell, Bouton & Frohardt, 2003; see also De Houwer, Beckers & Glautier, 2002). In the Blocking phase of the experiment, two new compounds BX and DY, both predicting –20 hormone level change, were introduced. We also included three familiar cues from the training phase (F0, G+20, GH+40) to maintain continuity between the two phases. Throughout both the Training and Blocking phases, cues B and D received identical training histories, and differed only as a function of whether they were part of a FN discrimination. Similarly, cues X and Y were both novel in the blocking phase of the study and were presented in compound with another familiar cue; both compounds were paired with a decrease in hormone level of the same magnitude.

**Table 3:** Design of Experiment 2.


TRAINING	BLOCKING PHASE	TEST PREDICTIONS	CAUSAL RATINGS	FORCED-CHOICE CAUSAL STRUCTURE ASSESSMENT

A+20 AB0	BX-20	A B AB	A B	B
C-20	DY-20	C	C	
DE0		DE D E	D E	
F0	F0			
G+20 H+20	G+20	G GH	G H	
GH+40	GH+40	I X Y	I X Y	


*Note*: + = an increase in hormone level, 0 = no change, and – = a decrease. Numeric values indicate the magnitude of change. Column headings describe each phase of the experiment in sequence from left to right, beginning with the Training phase and ending with the Forced-choice causal structure assessment. All stimuli presented in each phase (and their associated outcomes) are denoted below the relevant column heading.

At test, we assessed predictive ratings for cues X and Y individually. If participants inferred an Opposite Causal structure for B, they might determine from training that B alone produced a –20 change in hormone level. A consequence of this inference is that when participants were subsequently presented with BX leading to –20 hormone level change, they would be able to infer that X had no additional impact on hormone level. In contrast, we expected greater learning about Y since D had not already been established as a predictor of hormone decrease. As a result, we would expect greater blocking, indexed by less negative ratings to X compared to Y, in the Opposite Causal subgroup compared to participants in all other subgroups. We additionally predicted that participants in the Opposite Causal subgroup would provide similar ratings to B and to C, a cue that had been presented alone and predicted a –20 change in hormone levels.

### Method

#### Participants

One-hundred and fifty participants recruited through Prolific (91 female, *M_age_* = 24.8, *SD* = 6.23) participated in this study in exchange for monetary payment (20 min at £6GBP/hr).

#### Apparatus & Stimuli

**Figure 5:**
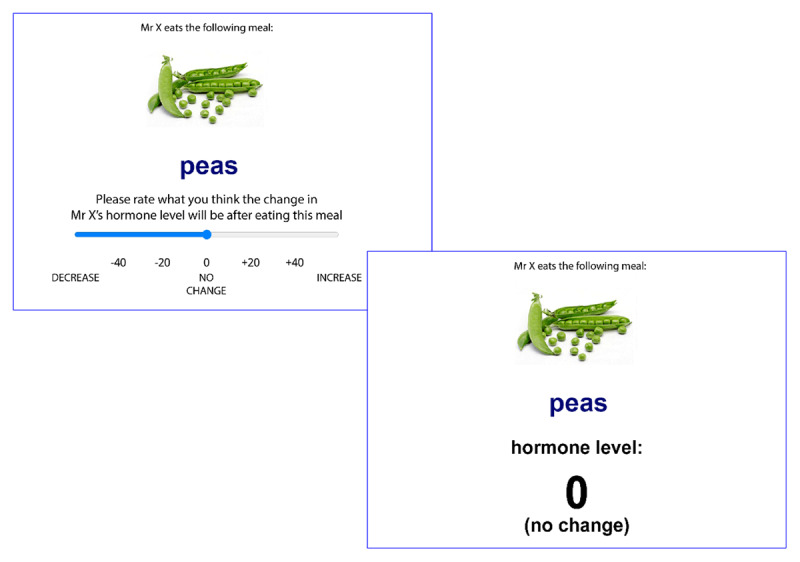
Example Screenshots from a Single Training Trial in Experiment 2, Where the Cue is Followed by No Change in Hormone Level.

A total of 11 cues were presented in this study, labelled as A–I, X and Y. X and Y were novel cues presented in the second phase of training as part of the blocking manipulation. Presentation of stimuli in this study was identical to that in Experiment 1, with the exception of the outcomes. Outcomes presented in this study consisted of numeric values from –20 to +40 in 20-point increments. Positive and negative signs were included to denote increases and decreases in hormone levels respectively (e.g. hormone level: +20). When there was no change in hormone level, a verbal description of the outcome was also presented in text below the numeric value (see Figure 5).

#### Procedure

The method used in this experiment was largely similar to Experiment 1 with the exception of an additional blocking phase. In the blocking phase, two novel compounds BX and DY were presented for the first time, both of which were followed by an outcome of –20. X and Y were also presented in test predictions and causal ratings at test. In the interest of saving time, we removed the open-ended assessment for B that was presented in Experiment 1.

Another point of difference between this experiment and Experiment 1 was the outcome prediction scale presented on each trial and in the test predictions. In this study, predictions were made on a scale from DECREASE to INCREASE with a mid-point of NO CHANGE. Numeric markers were also included on the scale from –40 to +40 with a mid-point of 0 aligning with the text description NO CHANGE. However, the extremes of the scale did not have a numeric value; this was to illustrate that the hormone level could decrease or increase by an indefinite value, and thereby avoid floor and ceiling effects (see De Houwer et al., 2002). Numeric markers were included to ensure that participants were learning the cue-outcome associations appropriately and made predictions that were consistent with outcomes they had been presented with. In both the Training and Blocking phase of the study, feedback on the change in hormone level was provided on each trial after participants had made a prediction. No feedback was provided at test, consistent with Experiment 1.

#### Statistical Analyses

In this experiment, we were primarily interested in the magnitude of the blocking effect determined by participants’ ratings to cue X compared to cue Y on both outcome predictions at test and causal ratings. Note that lower ratings to Y compared to X are indicative of greater blocking to X. We also compared participants’ ratings for cue B alone relative to the control cue D, as well as to the directly trained cue C, as a function of their reported causal structure subgroup in both outcome test predictions and causal ratings. Predictive ratings from the training phase were analysed using planned within-subject contrasts that tested average ratings for 1) stimuli that predicted a +20 outcome increase (G, H and A) compared to no change (AB and DE; +20 vs 0), 2) the stimulus predicting a +40 outcome increase (GH) vs the average of standard predictive cues (40 vs +20), 3) stimuli followed by no change compared to stimulus C which was followed by hormone level decrease (0 vs –20), 4) linear trend over six presentations, and 5) the interactions between contrasts 1–3 and linear trend of presentation. A similar set of contrasts was tested in the blocking phase, in addition to a comparison of participants’ ratings to BX and DY on the first trial of the blocking phase (prior to any feedback). For all contrasts described above, we also tested the interaction with causal structure subgroup using the same between-subjects contrasts as Experiment 1.

### Results

#### Exclusion criteria

The same exclusion criteria were used in this study as in Experiment 1. Of the 150 participants who completed the study, 11 were excluded for writing down information during the experiment, 5 were excluded for failing the instruction check more than twice, and an additional 16 participant was excluded for failing to meet the training criterion. After applying all the exclusions, 118 participants remained.

#### Causal structure assessment

Based on participants’ responses on the 4AFC question, we had 37 participants reporting a Configural structure, 22 reporting a Modulation structure, 23 reporting a Direct Prevention causal structure, and 36 reporting an Opposite Causal structure.

#### Training

**Figure 6:**
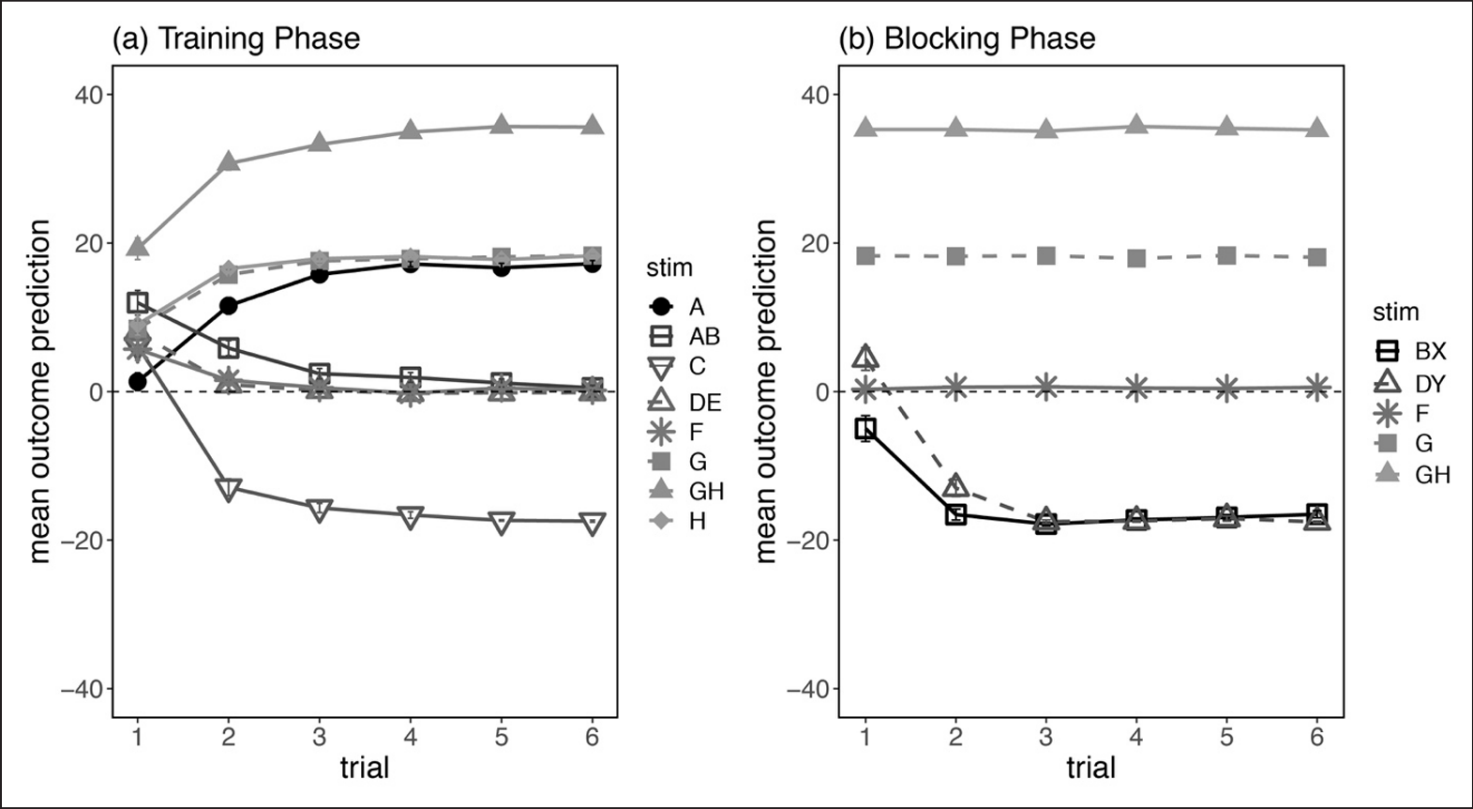
Mean Outcome Prediction (±SE) During **(a)** Training and **(b)** Blocking Phase in Experiment 2. *Note*: Filled symbols denote trials that were paired with a hormone level increase (e.g., G+20 and GH+40), and unfilled symbols denote trials that were followed by no change (F0) or by a hormone level decrease (e.g., BX-20 and DY-20).

Figure 6a illustrates mean prediction ratings across each presentation of the different trial types presented in the training phase average across all participants. No main effect or interaction contrasts involving causal structure were significant on this measure, all *F*s < 1. Analysis of stimuli predicting hormone level +20 compared to stimuli predicting no change showed a main effect of cue type, *F*(1,114) = 1849, *p* < .001, η_p_^2^ = .942, which interacted significantly with linear trend of presentation, *F*(1,114) = 259.3, *p* < .001, η_p_^2^ = .695. Similarly, there was a significant main effect of cue type when comparing standard predictive (+20) to the additivity predictive cues (+40), *F*(1,114) = 1417, *p* < .001, η_p_^2^ = .926; this also interacted significantly with linear trend of presentation, *F*(1,114) = 7.46, *p* = .007, η_p_^2^ = .061. Finally we found a main effect of cue type comparing stimuli leading to no change versus the reference cue C that predicted a decrease in hormone level (0 vs –20), *F*(1,114) = 948.5, *p* < .001, η_p_^2^ = .893, which also interacted with linear trend of presentation, *F*(1,114) = 59.6, *p* < .001, η_p_^2^ = .343. There was no main effect of linear trend, *F*(1,114) = 1.53, *p* = .219, η_p_^2^ = .013. Overall, these results confirm that across the six presentations, participants learned which outcomes were associated with each trial type, and there were no differences in acquisition as a function of causal structure subgroup.

#### Blocking phase

Figure 6b illustrates participants’ mean outcome predictions across six presentations of each trial type collapsed across causal structure subgroups. Contrasts comparing standard predictive cue, G+20, to the cue that was followed by no change (F0), revealed a main effect of cue type, *F*(1,114) = 4146, *p* < .001, η_p_^2^ = .973. There was also a main effect of cue type when we compared standard vs additivity predictive cues (G+20 vs GH+40), *F*(1,114) = 19909, *p* < .001, η_p_^2^ = .994, and when we compared the non-predictive cue to the blocked cues (0 vs –20), *F*(1,114) = 1420, *p* < .001, η_p_^2^ = .926. The effect of 0 vs –20 interacted significantly with linear trend across trials, *F*(1,114) = 148.9, *p* < .001, η_p_^2^ = .566. There was also an overall linear trend, *F*(1,114) = 117.2, *p* < .001, η_p_^2^ = .507, driven by the sharp decrease in ratings for cues BX and DY across trials. No other interaction contrasts, including main effect and interaction contrasts involving causal structure subgroups, reached statistical significance, largest *F*(1,114) = 1.71, *p* = .194, η_p_^2^ = .015.

Analysis of participants’ first rating on BX and DY trials revealed an overall main effect of cue type, *F*(1,114) = 15.6, *p* < .001, η_p_^2^ = .120, driven by higher ratings to DY than BX. However, this difference did not interact with any of the causal structure comparisons. Ratings in the Blocking phase broken down by causal structure subgroup can be found in Supplemental Materials. No other main effects or interactions were significant, *F*s < 1.

#### Outcome prediction at test

**Figure 7:**
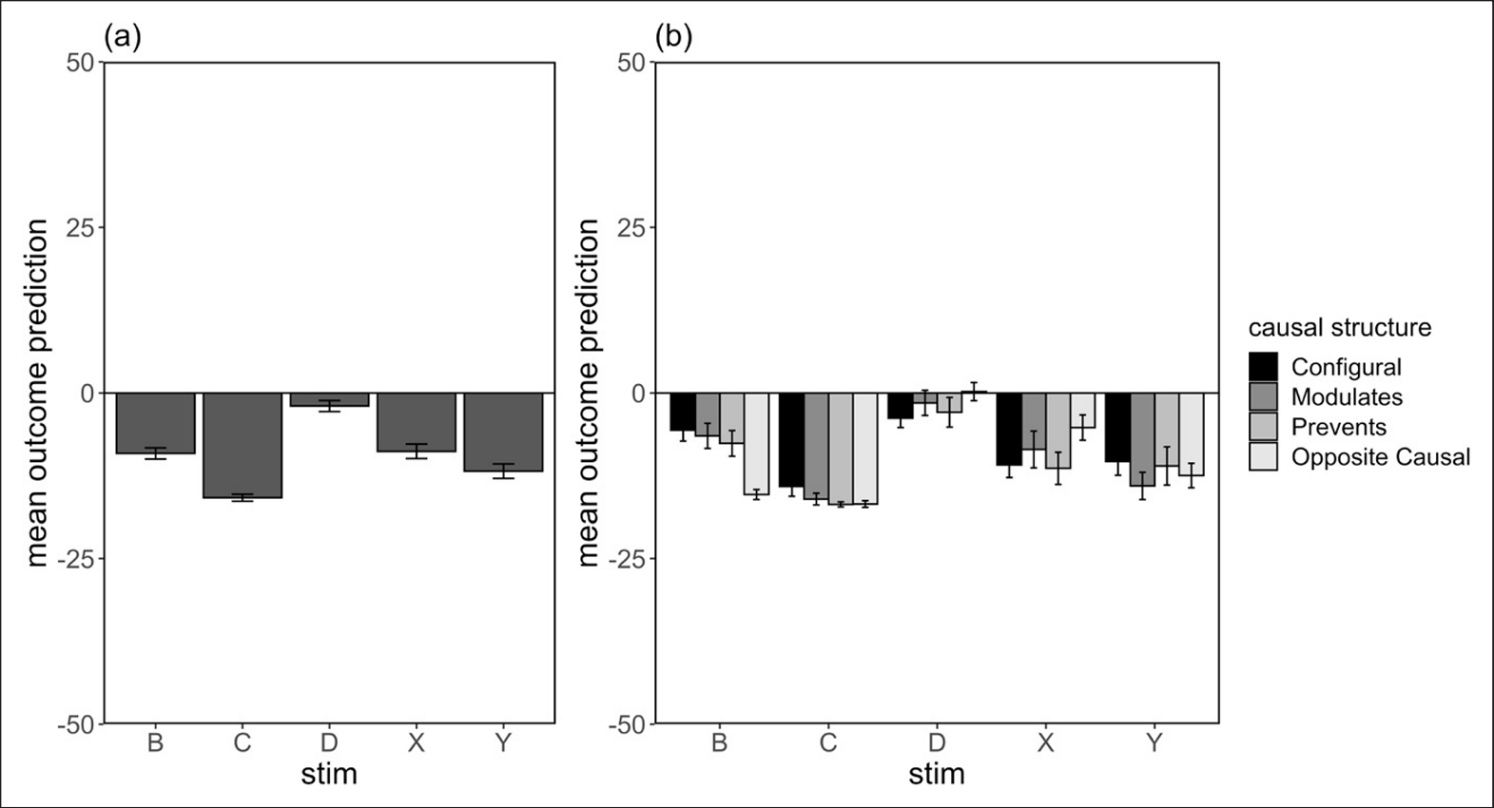
Average Outcome Prediction at Test (±SE) For Critical Stimuli **(a)** as a Group Average, and **(b)** Separated By Causal Structure Subgroup in Experiment 2.

Participants’ test predictions were averaged across the two presentations of each trial type. Figure 7 illustrates the mean outcome predictions for the cues of interest and their relevant controls, B, C, D, X and Y, averaged across all participants (7a) and separated by causal structure subgroup (7b). We were primarily interested in whether Opposite Causal participants show greater blocking of X relative to Y, indexed by less negative ratings to X, compared to all other subgroups. We additionally predicted that this subgroup of participants would provide equivalent (negative) prediction ratings to B alone and to a cue that directly predicted hormone level –20.

The comparison of ratings for X and Y showed a main effect of cue type, *F*(1,114) = 6.45, *p* = .012, η_p_^2^ = .054, with more negative ratings to Y relative to X. This is evidence of an overall blocking effect. There was also an interaction between cue type (X vs Y) and the contrast comparing Opposite Causal to all other subgroups, *F*(1,114) = 5.27, *p* = .023, η_p_^2^ = .044, indicative of a greater blocking effect in the Opposite Causal subgroup. No other main effects or interactions involving causal structure subgroup were significant, largest *F*(1,114) = 1.31, *p* = .256, η_p_^2^ = .011. Simple effects comparing the difference in ratings to X and Y for the Opposite Causal subgroup only showed a significant effect of cue type, *F*(1,35) = 19.1, *p* < .001, η_p_^2^ = .353.

Comparison of participants’ ratings to B relative to D also showed an overall effect of cue type, *F*(1,114) = 40.3, *p* < .001, η_p_^2^ = .261, which interacted significantly with causal structure contrasts comparing Opposite Causal to all other subgroups, *F*(1,114) = 26.7, *p* < .001, η_p_^2^ = .189. These results are similar to the finding from Experiment 1 that the Opposite Causal subgroup showed a stronger prediction that B would decrease hormone level compared to the other subgroups. Similarly, comparison of prediction ratings to B vs C, a cue which was presented alone followed by –20 hormone level, revealed a significant main effect of cue type, *F*(1,114) = 59.5, *p* < .001, η_p_^2^ = .343, and a significant interaction with causal structure contrasts comparing Opposite Causal to all other subgroups, *F*(1,114) = 14.9, *p* < .001, η_p_^2^ = .116. These results suggest that ratings to B were more similar to C for the Opposite Causal participants compared to all other subgroups. We additionally found significant main effect contrasts where Opposite Causal participants gave significantly lower ratings compared to all other subgroups collapsed across cues B and C, *F*(1,114) = 22.9, *p* < .001, η_p_^2^ = .167. Simple effects comparing ratings to B vs C for the Opposite Causal subgroup alone also showed no significant difference between ratings for the two cues, *F*(1,114) = .780, *p* = .379, η_p_^2^ = .007. Together, these results suggest that ratings to B were much more negative, and were in fact equivalent to ratings to C, for participants in the Opposite Causal subgroup compared to all other subgroups; these results are consistent with our predictions. Finally, participants in the Configural subgroup also gave significantly less negative ratings compared to all other subgroups averaged across both cues, *F*(1,114) = 10.4, *p* = .002, η_p_^2^ = .084. No other analysis involving causal structure subgroup was significant, *F* < 1.

#### Causal ratings

**Figure 8:**
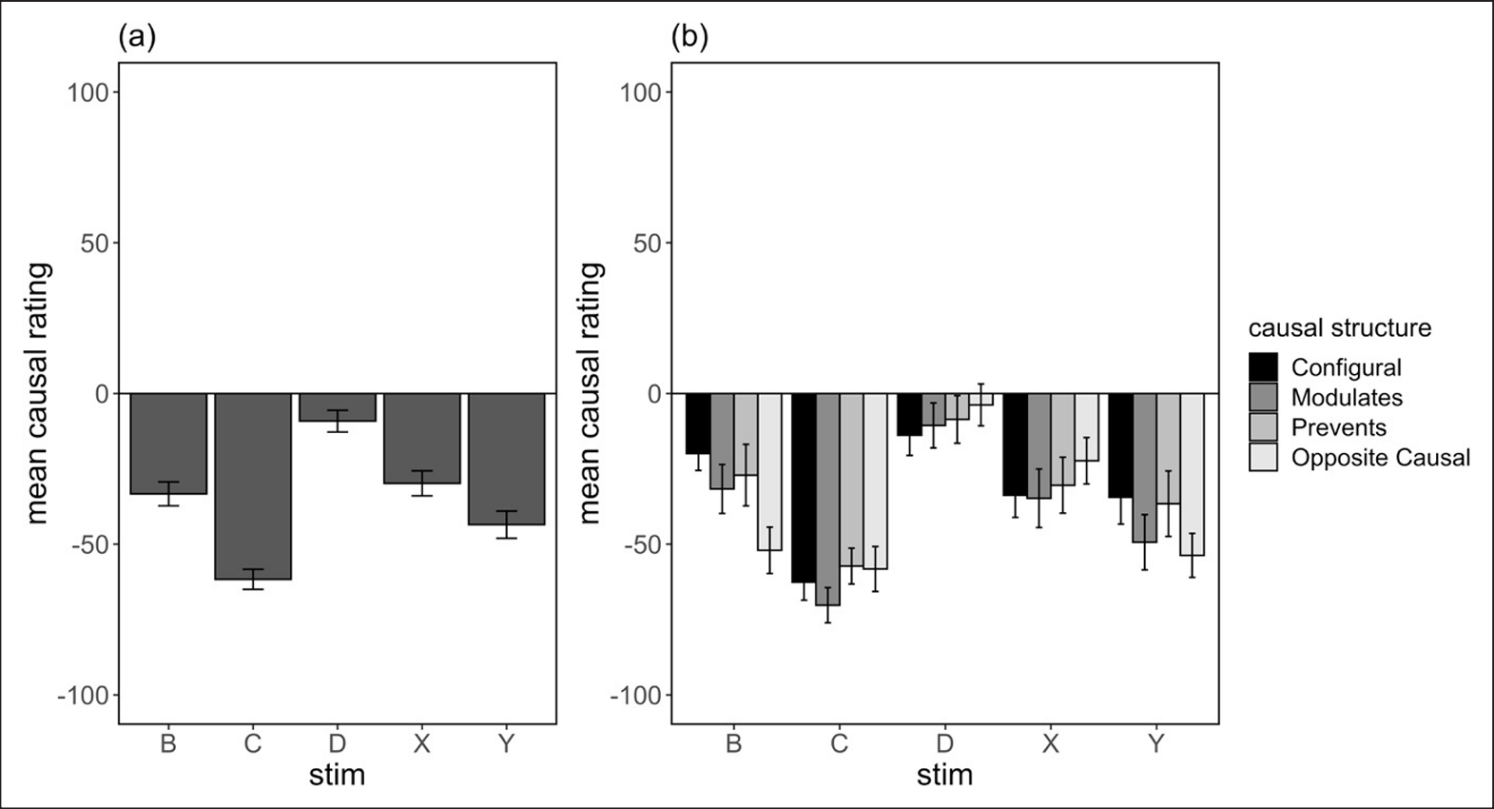
Mean Causal Ratings at Test (±SE) for Critical Cues **(a)** as a Group Average, and **(b)** Separated by Causal Structure Subgroup in Experiment 2.

Mean causal ratings for cues B, C, D, X and Y are shown in Figure 8 as a group average (8a) and separated by causal structure subgroups (8b). Consistent with the outcome prediction ratings, analysis of causal ratings for X and Y showed a main effect of cue, *F*(1,114) = 6.07, *p* = .015, η_p_^2^ = .050, which interacted significantly with the contrast comparing Opposite Causal to all other subgroups, *F*(1,114) = 4.53, *p* = .035, η_p_^2^ = .038. This finding confirms stronger blocking of learning that X predicted a decrease in hormone level. Simple effects comparing X and Y for the Opposite Causal subgroup only showed a significant difference in ratings, *F*(1,35) = 14.0, *p* < .001, η_p_^2^ = .286. No other main effects of interactions involving causal structure subgroups were significant, *F*s < 1.

Comparison of causal ratings to B vs D showed a similar pattern of results to the test predictions; there was a main effect of cue type, *F*(1,114) = 20.0, *p* < .001, η_p_^2^ = .149, which interacted significantly with contrasts comparing Opposite Causal to all other subgroups, *F*(1,114) = 8.72, *p* = .004, η_p_^2^ = .072. As in Experiment 1, the Opposite Causal subgroup gave stronger preventive ratings for B than the other subgroups. No other main effect or interactions involving causal structure contrasts were significant, largest *F*(1,114) = 2.41, *p* = .124, η_p_^2^ = .021.

Similar comparison of causal ratings to B vs C produced the same pattern of results, with a main effect of cue type, *F*(1,114) = 40.8, *p* < .001, η_p_^2^ = .264, which interacted significantly with contrasts comparing Opposite Causal to all other subgroups, *F*(1,114) = 9.99, *p* = .002, η_p_^2^ = .008. No other main effects or interactions were significant, largest *F*(1,114) = 3.34, *p* = .070, η_p_^2^ = .028. Simple effects comparing ratings to B vs C for the Opposite Causal subgroup only revealed no significant difference in causal ratings to the two cues, *F*(1,114) = .586, *p* = .445, η_p_^2^ = .005. These findings present confirmatory evidence that participants who reported an Opposite Causal structure treated the feature B as equivalent to a cue that independently produced a decrease in hormone level. Importantly, this pattern of results was significantly different to all other subgroups, including the two inhibitory subgroups (see Figure 8b).

### Discussion

The results from Experiment 2 extended the findings from Experiment 1 to a blocking manipulation, where the feature in a FN discrimination blocked learning of a novel cue paired with hormone level decrease in a subsequent phase. Outcome test predictions for X compared to Y revealed a significant overall blocking effect, with more negative ratings (indicating hormone level decrease) to the control cue than to the blocked cue. Importantly, the magnitude of the blocking effect was greater for participants who endorsed an Opposite Causal structure than for the other subgroups. These subgroup differences were also found when we compared predictions to B relative to D, with greater negative predictions to B in the Opposite Causal subgroup. This subgroup difference for B compared to D was also obtained in the causal ratings. We additionally showed that participants who reported an Opposite Causal structure gave ratings to B similar to those of a cue that directly led to a hormone level decrease (C–20). This is further evidence that when presented with a FN discrimination, some participants infer that B alone directly led to the opposite outcome, and treated B as equivalent to a cue that was explicitly paired with hormone level decrease.

One potential criticism of the design of Experiment 2 is in the introduction of the additivity compound GH+40, where each element G and H were separately paired with an outcome of +20. The inclusion of this additivity compound might encourage participants to solve the FN discrimination by calculating the arithmetic difference between the outcome associated with A alone (+20) and the no change outcome on AB trials, which might then lead them to infer that B directly causes a decrease in hormone level. However, it should be noted that this arithmetic summation process is consistent with Melchers et al.’s (2006) argument that bidirectional outcomes mirror the symmetrical continuum of associative strengths assumed by the Rescorla-Wagner model, and should therefore encourage direct prevention learning. In contrast, our results suggest an alternative explanation that the discrepancy between A and AB trials encourages excitatory learning of B leading to the opposite outcome, indexed by the ability for B alone to generate predictions of hormone level decrease and block subsequent learning to X.

## General Discussion

In this study, we were interested in testing the possibility that using bidirectional outcomes in an A+/AB– feature negative task allows participants to acquire an opposite excitatory causal structure to B that is distinct from known inhibitory structures like direct prevention and modulation. Both experiments showed that some participants inferred an Opposite Causal structure when presented with an outcome that could either increase or decrease. In Experiment 1, we showed that direct experience with a negative outcome was not necessary for participants to infer an Opposite Causal structure, nor did it lead to more participants reporting this causal structure to B. It appears that using an outcome that has the potential to vary in both directions is sufficient to lead some participants to the conclusion that the feature B directly causes a decrease in hormone levels, even without having seen a negative outcome. This finding poses a challenge for previous studies that have used the presence of a negative outcome (hormone level decrease) to differentiate between bidirectional and unidirectional outcomes, particularly when changes in hormone level were from an unspecified baseline (e.g., Melchers et al., 2006; Lotz & Lachnit, 2009; Baetu & Baker, 2010). It is plausible that in these studies, the manipulation used to establish outcome directionality was not effective, and that differences between groups at test were a product of extraneous factors, such as differences in the scales presented during training vs test (e.g., Baetu & Baker, 2010) or differences in scales presented for the unidirectional vs bidirectional outcome groups (e.g., Melchers et al., 2006; Lotz & Lachnit, 2009).

We also found evidence of a subgroup difference in Experiment 1, where participants in the Opposite Causal subgroup showed the greatest transfer of B’s properties to a novel cause in a summation test (i.e., greater CB–CD difference) compared to all other subgroups. We have previously proposed that inhibitory summation is best thought of as an instance of generalisation (Chow et al., 2022; see also Bonardi, Robinson & Jennings, 2017). Specifically, we suggested that in a FN discrimination task with *uni*directional outcomes, individual differences in inhibitory transfer between Modulators and Preventers may not reflect qualitative differences in what is learned (i.e., occasion-setting vs direct prevention), but differences in participants’ willingness to generalise inhibitory properties of the feature from training to test. In the present study, participants who endorsed an Opposite Causal structure were perhaps solving the FN discrimination differently to the Direct Prevention and Modulation subgroups. That is, they learned that B by itself directly produces a reduction in hormone level. This causal structure is straightforward to apply to a summation test since it does not involve generalisation—B’s relationship with the outcome is independent of A. Thus, prediction ratings to CB are low since the expectation of hormone level increase in the presence of C is offset by the strong expectation of hormone level decrease to B, with minimal generalisation decrement in the summation test. The strong transfer seen among Opposite Causal participants together with strong preventative ratings to B alone in causal ratings compared to all other subgroups, provide further support that at least for some participants, causal learning about B with the opposite outcome has occurred.

In Experiment 2, we found further evidence of opposite causal learning with bidirectional outcomes, where the feature was again inferred to cause a decrease in hormone levels. Presenting B together with a novel cue X successfully blocked learning that X predicted hormone level decrease when the compound was directly paired with a hormone level decrease. These findings are diagnostic of opposite causal learning to B since no additional information was provided by directly pairing B with hormone decrease, hence leading to blocking of learning to X. Causal and predictive ratings to B alone were also similar in magnitude to cue C, which had been explicitly paired with hormone level decrease. These results suggest that for participants in the Opposite Causal subgroup, the feature B was treated as equivalent to a cue that was active in producing a reduction in hormone levels by itself. These findings are in contrast to established theories of inhibitory learning which involve the suppression of the outcome elicited by a causal/excitatory cue, either by modulating the effect of the excitor or by directly preventing the outcome from occurring. Consistent with these accounts, inhibitors are typically thought to be behaviourally silent (Rescorla, 1969), with few demonstrations where the inhibitor directly elicits behaviour (but see Wasserman, Franklin & Hearst, 1974).

In summary, our findings propose an alternative explanation to the idea proposed by Melchers et al (2006) that bidirectional outcomes in FN discrimination encourages direct prevention learning. We have shown that under these conditions, some participants are learning a generative causal relationship between the feature and the opposite outcome. This is distinct from an opposite *inhibitory* mechanism like the negative associative strength governing inhibition in the RW model, since in opposite causal learning the action of the feature is *direct* (i.e., does not require the presence of a background cause), and *generative* (does not involve the suppression of outcome activation). It is also important to note that the RW model in its original conception was designed to explain inhibitory learning with unidirectional outcomes, such as the presence or absence of a foot shock or food pellets in animal conditioning procedures. These outcomes may vary in magnitude, but they are unable to take on negative properties (i.e., anti-outcome). Thus, the model does not make any assumptions about the symmetry between the dimension of associative strength and the properties of the outcome, nor does it specify this as a requirement for the feature acquiring negative associative strength when an expected outcome is omitted. Importantly, any model that seeks to explain how inhibitory relationships are learned should not be limited to outcomes with a positive and negative dimension, but should account also for how we learn about discrete events where negative values are not possible. Indeed, it seems like there is nothing inherently special about a negative outcome that encourages learning distinct from that of causal learning in the positive direction. Negative outcomes are commonly used in human causal learning experiments, such as in a medical scenario where a drug cue is thought to lead to recovery from illness (e.g., Matute, Yarritu & Vadillo, 2011; Chow, Colagiuri & Livesey, 2019). These scenarios typically frame the relationship between treatment use and recovery as causal (treatment causes recovery); however recovery from illness may be more accurately construed as the reduction or termination of illness.

Returning to the issue we began this paper with, what exactly are people learning when they experience a negative relationship? We have previously shown that there are substantial individual differences in human inhibitory learning using a feature negative arrangement with a unidirectional outcome (Lee & Lovibond, 2021; see also Glautier & Brudan, 2019). Additionally, we found that self-reported causal structure was predictive of the degree of transfer in a summation test. We have also suggested that differences in inhibitory transfer between Direct Prevention and Modulation subgroups is a result of differences in willingness to generalise inhibitory properties of the feature from training to test, rather than qualitative differences in what is learned (Chow et al., 2022). The novel contribution of the current paper is to present another possible causal structure in a FN procedure, which arises when a bidirectional outcome is used. We propose that rather than encouraging direct prevention (inhibitory) learning, as originally suggested by Melchers et al. (2006), bidirectional outcomes encourage at least some participants to learn an opposite *excitatory* causal structure. In our experiments, this subgroup of participants produced significantly different results to Configural, Direct Prevention and Modulation participants, showing greatest transfer in a summation test, greatest prediction of a decrease in hormone level when B was presented alone, and strongest blocking of a novel cue paired with a reduction in hormone level. Importantly, these participants did not report learning an inhibitory relationship between the feature and the outcome produced by the causal cue. Instead, they reported having formed a *generative* association between the feature and the opposite outcome. These findings suggest that evidence of a direct *prevention* structure is less prevalent than previously assumed. Together, these findings support our previous proposal that inhibitory learning in humans may perhaps be largely modulatory in nature (Lee & Lovibond, 2021).

In conclusion, we propose an alternative to the claim that FN discrimination with a bidirectional outcome encourages direct prevention learning. We have shown that FN discrimination with a bidirectional outcome can be solved by assuming a causal relationship between the feature and the opposite outcome to that signalled by the partner cue during FN training. Together with previous findings that most participants infer a modulatory causal structure when solving a FN discrimination with unidirectional outcomes, we recommend that more attention be given to alternative mechanisms of prevention learning such as modulation.

## Data Accessibility Statement

All data is made available on the Open Science Framework and can be accessed at https://osf.io/9at4b/. None of the experiments were pre-registered.

## Additional File

The additional file for this article can be found as follows:

10.5334/joc.266.s1 Supplemental Materials. Analyses and Figures.
