# Supplementary material for: Inhibitory Learning with Bidirectional Outcomes: Prevention Learning or Causal Learning in the Opposite Direction?
Source: J Cogn. 2023 Mar 10;6(1):19. doi: 10.5334/joc.266 (PMC10000320; doi:10.5334/joc.266)
Supplement: Supplemental Materials. — Analyses and Figures. [file joc-6-1-266-s1.pdf]

Supplemental Materials

S1.1 Screenshot of instruction check prior to starting experiment

Check your knowledge before you begin!

Question 1: On each trial, your task is to make a prediction about what Mr X's hormone level will be given that he has eaten certain foods.*

○ TRUE
○ FALSE

Question 2: You will have to do this through a process of trial and error, but you will receive feedback to help you learn. *

○ TRUE
○ FALSE

Question 3: You will only see each food once. *

○ TRUE
○ FALSE

Continue

S1.2 Results of contrasts involving causal structure subgroups

Experiment 1

Training

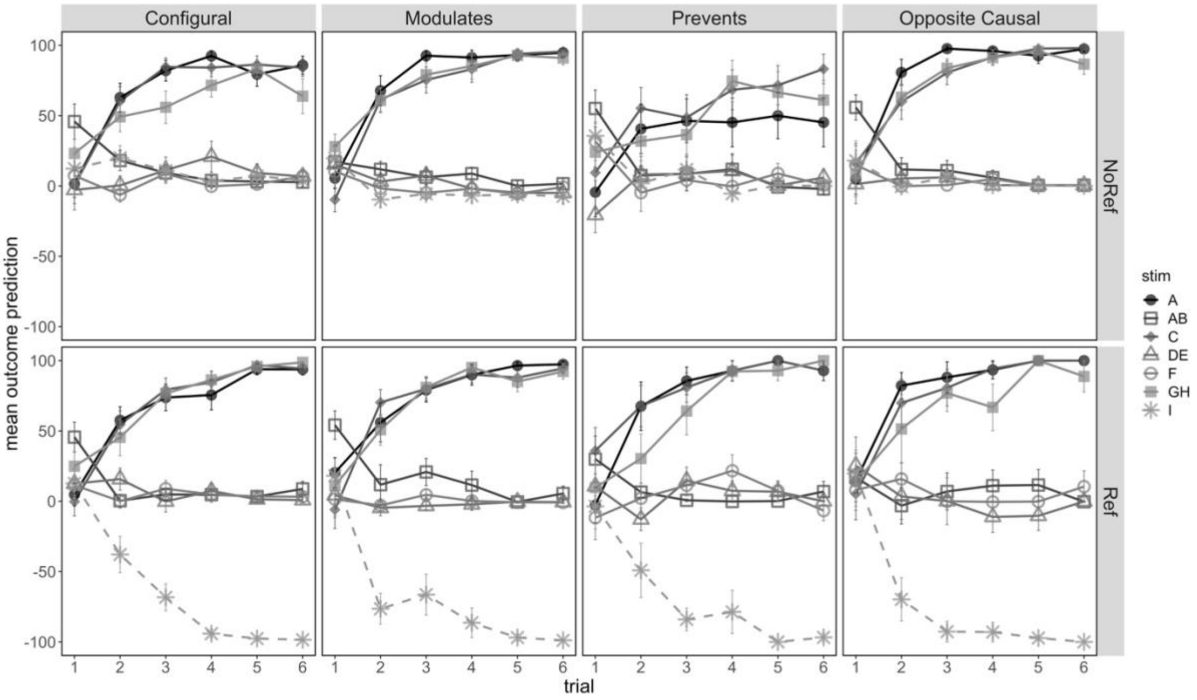

Opposite Causal vs Others

- Linear trend, $F(1,133) = .047$, $p = .828$, $\eta_p^2 < .001$
- predictive vs non-predictive cues, $F(1,133) = 5.03$, $p = .027$, $\eta_p^2 = .036$
- linear x predictive vs non-predictive cues, $F(1,133) = .547$, $p = .461$, $\eta_p^2 = .004$
- reference cue, $F(1,133) = .287$, $p = .593$, $\eta_p^2 = .002$
- reference cue x linear trend, $F(1,133) = .303$, $p = .583$, $\eta_p^2 = .002$

Group x Opposite Causal, $F(1,133) = 1.74$, $p = .190$, $\eta_p^2 = .013$

- Linear trend, $F(1,133) = .350$, $p = .555$, $\eta_p^2 = .003$
- predictive vs non-predictive cues, $F(1,133) = 1.14$, $p = .288$, $\eta_p^2 = .008$
- linear x predictive vs non-predictive cues ,$F(1,133) = .961$, $p = .329$, $\eta_p^2 = .007$
- reference cue, $F(1,133) = .087$, $p = .768$, $\eta_p^2 = .001$
- reference cue x linear trend, $F(1,133) = .235$, $p = .629$, $\eta_p^2 = .002$

Configural vs Inhibitory

- Linear trend, $F(1,133) = .008$, $p = .928$, $\eta_p^2 < .001$
- predictive vs non-predictive cues, $F(1,133) = .201$, $p = .655$, $\eta_p^2 = .002$
- linear x predictive vs non-predictive cues, $F(1,133) = .005$, $p = .945$, $\eta_p^2 < .001$
- reference cue, $F(1,133) = .621$, $p = .432$, $\eta_p^2 = .005$
- reference cue x linear trend, $F(1,133) = .137$, $p = .712$, $\eta_p^2 = .001$

Group x Configural vs Inhibitory, $F(1,133) = .910$, $p = .342$, $\eta_p^2 = .007$

- Linear trend, $F(1,133) = 2.79$, $p = .097$, $\eta_p^2 = .021$
- predictive vs non-predictive cues, $F(1,133) = .910$, $p = .342$, $\eta_p^2 = .007$
- linear x predictive vs non-predictive cues, $F(1,133) = .754$, $p = .387$, $\eta_p^2 = .006$
- reference cue, $F(1,133) = .083$, $p = .773$, $\eta_p^2 = .001$
- reference cue x linear trend, $F(1,133) = .532$, $p = .467$, $\eta_p^2 = .004$

<u>Test Predictions</u>

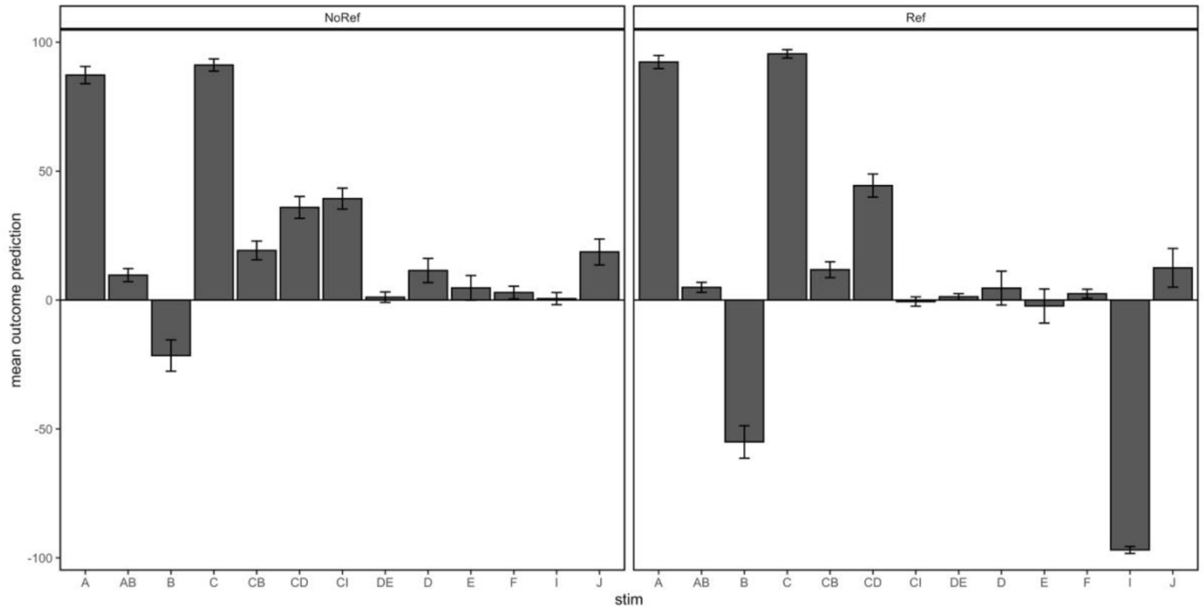

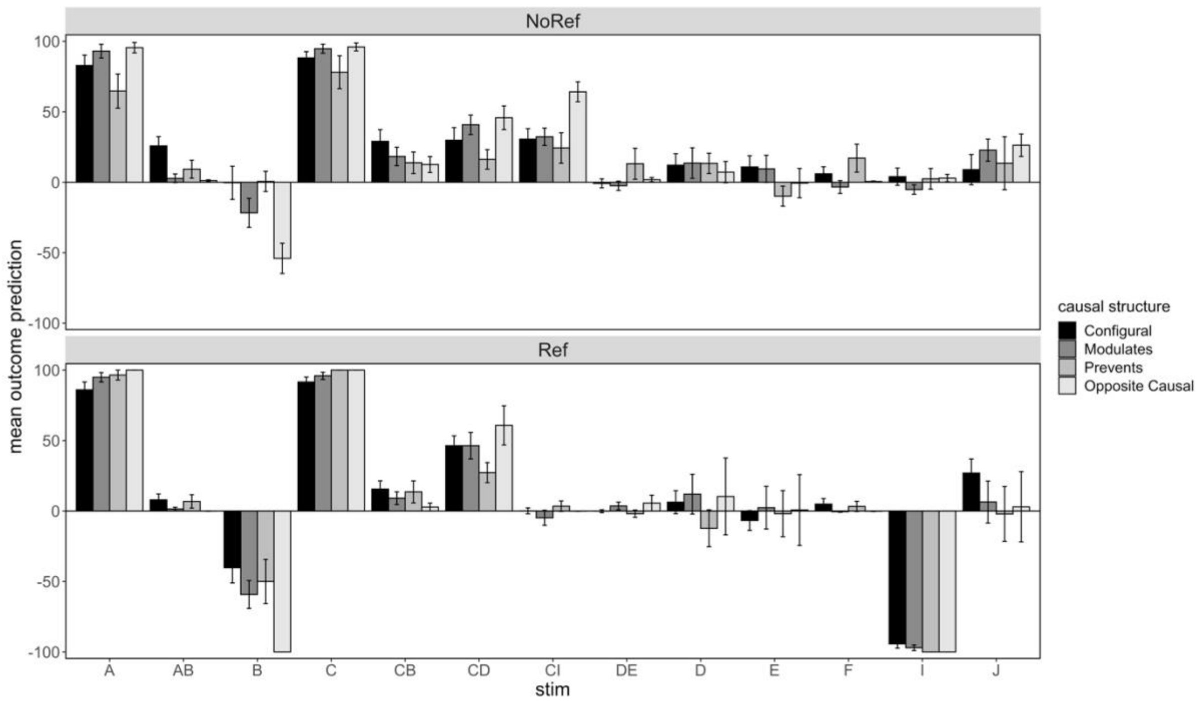

Summation Test (CB vs CD)

Opposite Causal vs Other

CB vs CD intx, $F(1,133) = 8.68$, $p = .004$, $\eta_p^2 = .061$

Main effect, $F(1,133) = .776$, $p = .380$, $\eta_p^2 = .006$

Group x Opposite Causal vs Other

CB vs CD intx, $F(1,133) = .105$, $p = .746$, $\eta_p^2 < .001$

Main effect, $F(1,133) = .006$, $p = .937$, $\eta_p^2 < .001$

Configural vs inhibitory

CB vs CD intx, $F(1,133) = .151$, $p = .699$, $\eta_p^2 = .001$

Main effect, $F(1,133) = 2.03$, $p = .157$, $\eta_p^2 = .015$

Group x Configural vs inhibitory

CB vs CD intx, $F(1,133) = 1.08$, $p = .302$, $\eta_p^2 = .008$

Main effect, $F(1,133) = .001$, $p = .982$, $\eta_p^2 < .001$

Cue B alone

Opposite Causal vs Other

Main effect, $F(1,133) = 19.4$, $p < .001$, $\eta_p^2 = .127$

Group interaction, $F(1,133) = .023$, $p = .881$, $\eta_p^2 < .001$

Configural vs Inhibitory

Main effect, $F(1,133) = 1.64$, $p = .203$, $\eta_p^2 = .012$

Group interaction, $F(1,133) = .048$, $p = .826$, $\eta_p^2 = .004$

Cue I alone

Opposite Causal vs Other

Main effect, $F(1,133) = .002$, $p = .962$, $\eta_p^2 < .001$

Group interaction, $F(1,133) = .555$, $p = .458$, $\eta_p^2 = .004$

Configural vs Inhibitory

Main effect, $F(1,133) = 2.26$, $p = .135$, $\eta_p^2 = .017$

Group interaction, $F(1,133) = .029$, $p = .865$, $\eta_p^2 < .001$

Causal Ratings

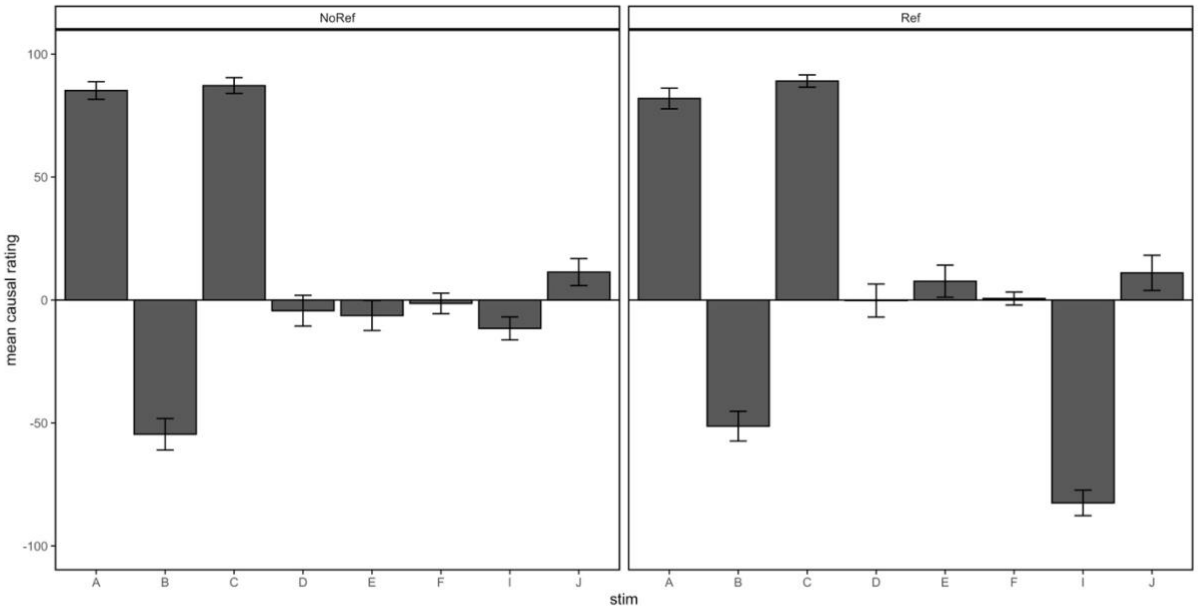

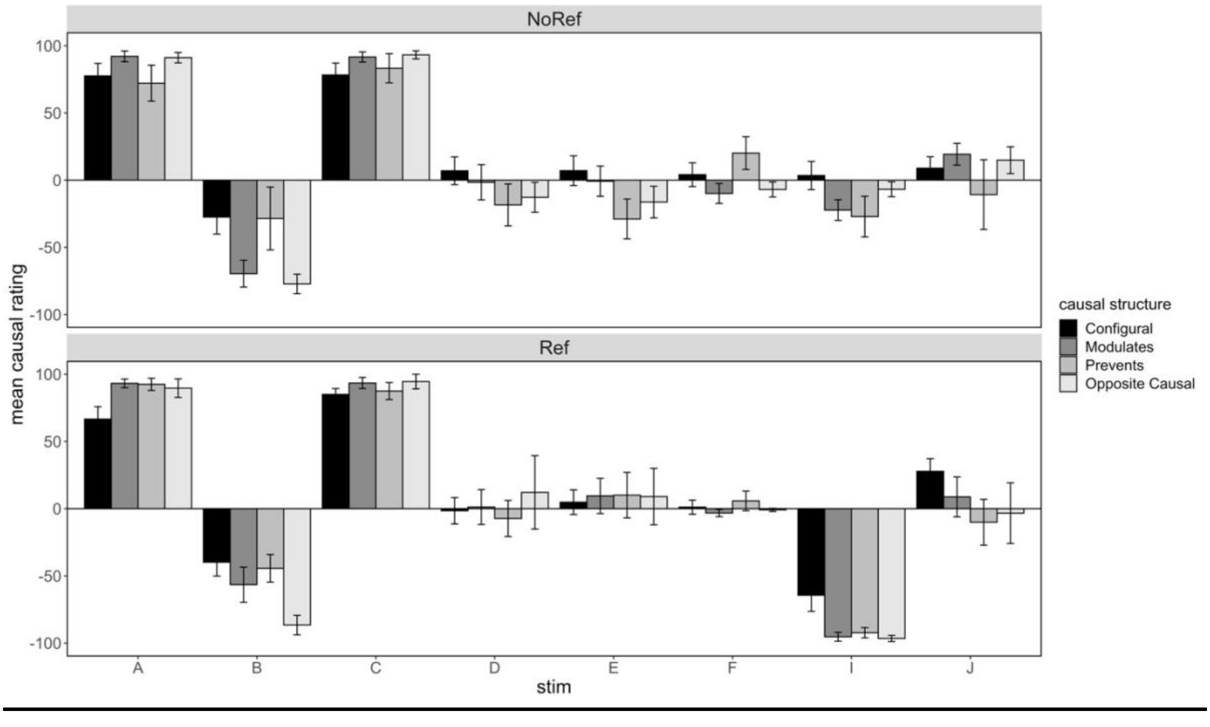

B vs D

Opposite Causal vs Other

Main effect, $F(1,133) = 4.72$, $p = .032$, $\eta_p^2 = .034$

x B vs D intx, $F(1,133) = 5.27$, $p = .023$, $\eta_p^2 = .038$

x Group, $F(1,133) = .352$, $p = .554$, $\eta_p^2 = .003$

x Group x B vs D, $F(1,133) = .600$, $p = .440$, $\eta_p^2 = .004$

Configural vs Inhibitory

Main effect, $F(1,133) = 3.36$, $p = .069$, $\eta_p^2 = .025$

x B vs D intx, $F(1,133) = .198$, $p = .657$, $\eta_p^2 = .001$

X Group, $F(1,133) = .923$, $p = .338$, $\eta_p^2 = .007$

x Group x B vs D, $F(1,133) = .021$, $p = .884$, $\eta_p^2 < .001$

Cue I alone

Opp Causal vs Others

Main effect, $F(1,133) = .049$, $p = .825$, $\eta_p^2 < .001$

Group interaction, $F(1,133) = 1.36$, $p = .246$, $\eta_p^2 = .010$

Configural vs Inhibitory

Main effect, $F(1,133) = 13.2$, $p < .001$, $\eta_p^2 = .090$

Group interaction, $F(1,133) = .007$, $p = .935$, $\eta_p^2 < .001$

Experiment 2 Results

Training Phase 1

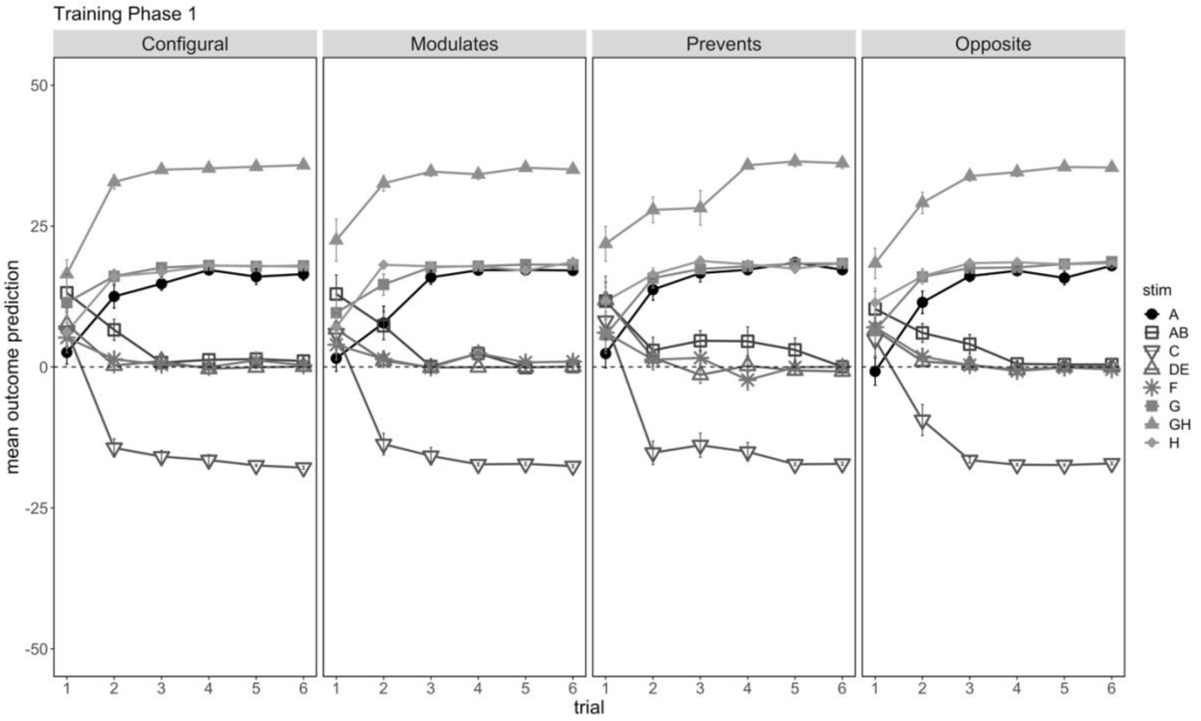

Opposite vs Others,

Main effect 20vs0, $F(1,114) = .143$, $p = .706$, $\eta_p^2 = .001$

Main effect 40vs20, $F(1,114) = .419$, $p = .519$, $\eta_p^2 = .004$

Main effect 0 vs -20, $F(1,114) = .173$, $p = .678$, $\eta_p^2 = .002$

Linear trend, $F(1,114) = .188$, $p = .665$, $\eta_p^2 = .002$

Linear x 20 vs 0, $F(1,114) = .221$, $p = .639$, $\eta_p^2 = .002$

Linear x 40 vs 20, $F(1,114) = .059$, $p = .808$, $\eta_p^2 = .001$

Linear x 0 vs -20, $F(1,114) = .005$, $p = .946$, $\eta_p^2 < .001$

Configural vs Inhibitory,

Main effect 20vs0, $F(1,114) = .040$, $p = .841$, $\eta_p^2 < .001$

Main effect 40vs20, $F(1,114) = .077$, $p = .782$, $\eta_p^2 = .001$

Main effect 0 vs -20, $F(1,114) = .131$, $p = .718$, $\eta_p^2 = .001$

Linear trend, $F(1,114) = .180$, $p = .672$, $\eta_p^2 = .002$

Linear x 20 vs 0, $F(1,114) = .344$, $p = .559$, $\eta_p^2 = .003$

Linear x 40 vs 20, $F(1,114) = .982$, $p = .324$, $\eta_p^2 = .009$

Linear x 0 vs -20, $F(1,114) = .010$, $p = .920$, $\eta_p^2 < .001$

Blocking Phase

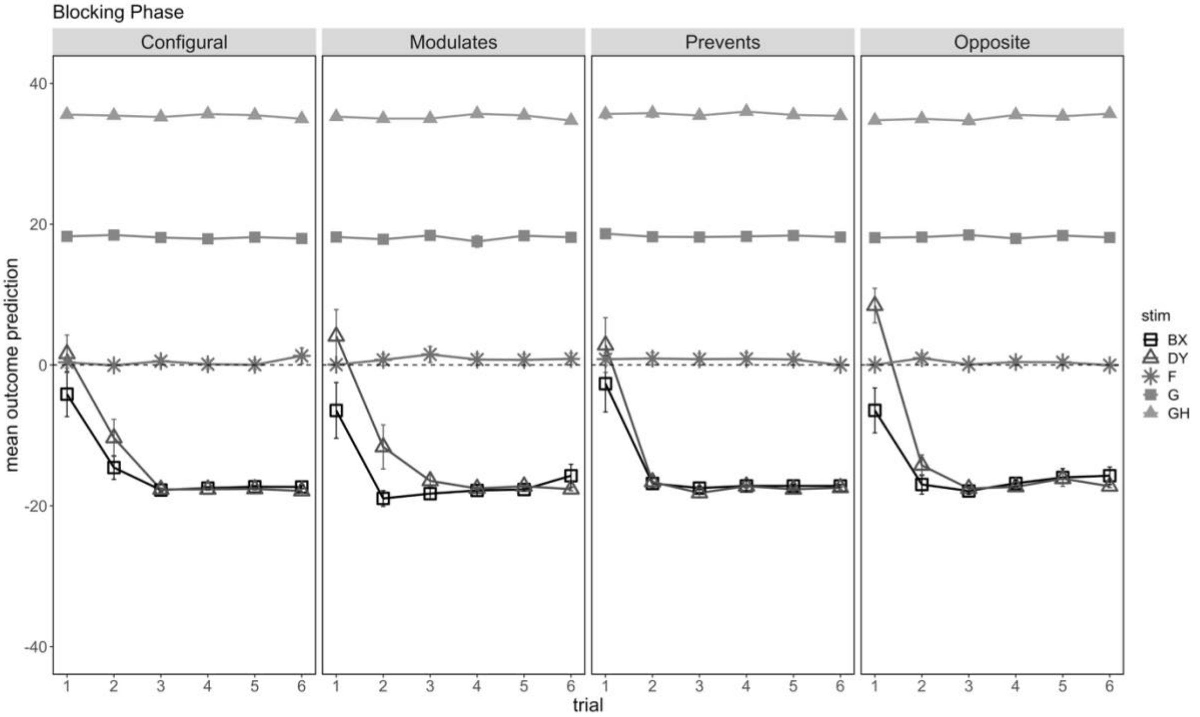

Opposite vs Others,

Main effect 20vs0, $F(1,114) = .315$, $p = .576$, $\eta_p^2 = .003$

Main effect 40vs20, $F(1,114) = .997$, $p = .320$, $\eta_p^2 = .009$

Main effect 0 vs -20, $F(1,114) = 1.15$, $p = .287$, $\eta_p^2 = .010$

Linear trend, $F(1,114) = .246$, $p = .621$, $\eta_p^2 = .002$

Linear x 20 vs 0, $F(1,114) = .679$, $p = .412$, $\eta_p^2 = .006$

Linear x 40 vs 20, $F(1,114) = 1.71$, $p = .194$, $\eta_p^2 = .015$

Linear x 0 vs -20, $F(1,114) = .083$, $p = .774$, $\eta_p^2 = .001$

Configural vs Inhibitory,

Main effect 20vs0, $F(1,114) = .228$, $p = .634$, $\eta_p^2 = .002$

Main effect 40vs20, $F(1,114) = .009$, $p = .927$, $\eta_p^2 < .001$

Main effect 0 vs -20, $F(1,114) = .559$, $p = .456$, $\eta_p^2 = .005$

Linear trend, $F(1,114) = .186$, $p = .667$, $\eta_p^2 = .002$

Linear x 20 vs 0, $F(1,114) = 1.35$, $p = .248$, $\eta_p^2 = .012$

Linear x 40 vs 20, $F(1,114) = .012$, $p = .913$, $\eta_p^2 < .001$

Linear x 0 vs -20, $F(1,114) = .770$, $p = .382$, $\eta_p^2 = .007$

First trial of Blocking Phase, comparing BX to DY

Opposite vs others, $F(1,114) = 2.40$, $p = .124$, $\eta_p^2 = .021$
Configural vs Inhibitory, $F(1,114) = .177$, $p = .675$, $\eta_p^2 = .002$

Test Predictions

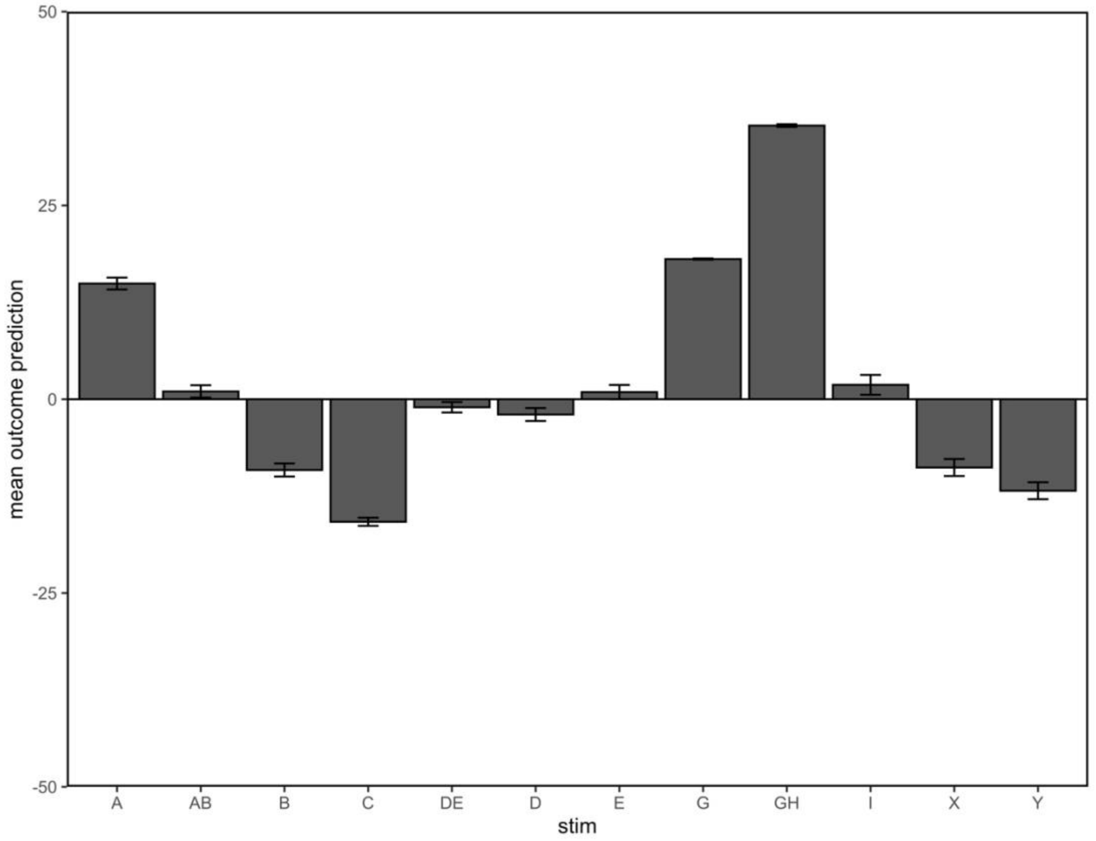

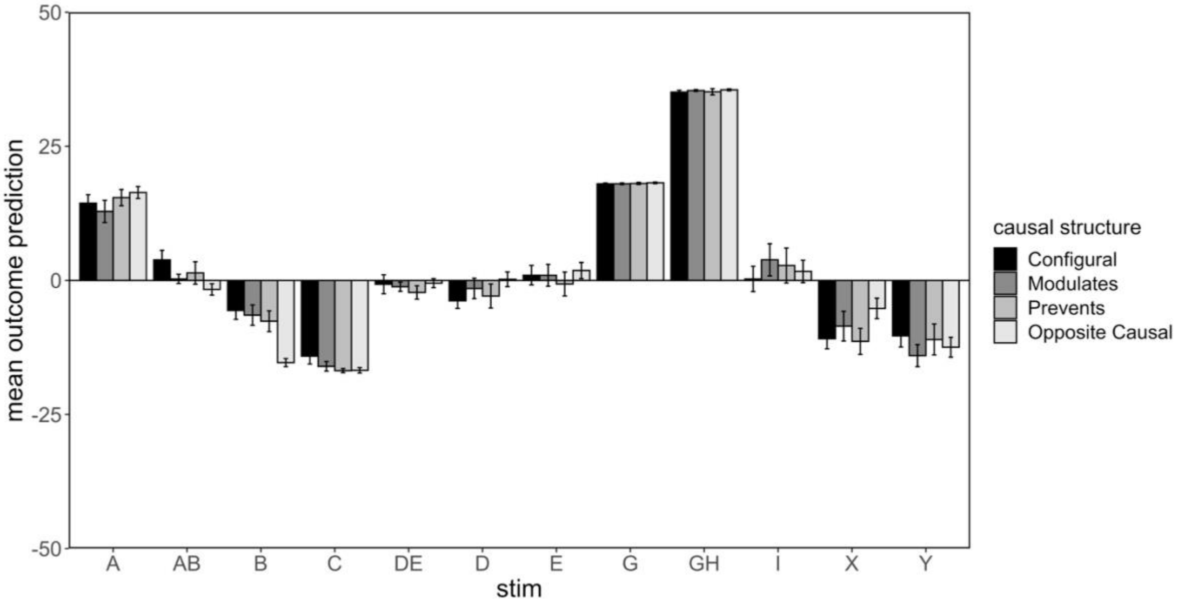

X vs Y

Opp vs Others

Main effect, $F(1,114) = 1.14$, $p = .288$, $\eta_p^2 = .010$,

X vs Y interaction, $F(1,114) = 5.27$, $p = .023$, $\eta_p^2 = .044$

Config vs Inhib

Main effect, $F(1,114) = .077$, $p = .782$, $\eta_p^2 = .007$

X vs Y interaction, $F(1,114) = 1.31$, $p = .256$, $\eta_p^2 = .011$

B vs D

Opposite vs Others

Main effect, $F(1,114) = 4.75$, $p = .031$, $\eta_p^2 = .040$

x B vs D interaction, $F(1,114) = 26.7$, $p < .001$, $\eta_p^2 = .189$

Configural vs Inhibitory

Main effect, $F(1,114) = .003$, $p = .958$, $\eta_p^2 < .001$

x B vs D interaction, $F(1,114) = 1.45$, $p = .232$, $\eta_p^2 = .013$

Cue B alone

Opposite vs Others, $F(1,114) = 27.4$, $p < .001$, $\eta_p^2 = .193$
Configural vs Inhib, $F(1,114) = .596$, $p = .442$, $\eta_p^2 = .005$

Causal Ratings

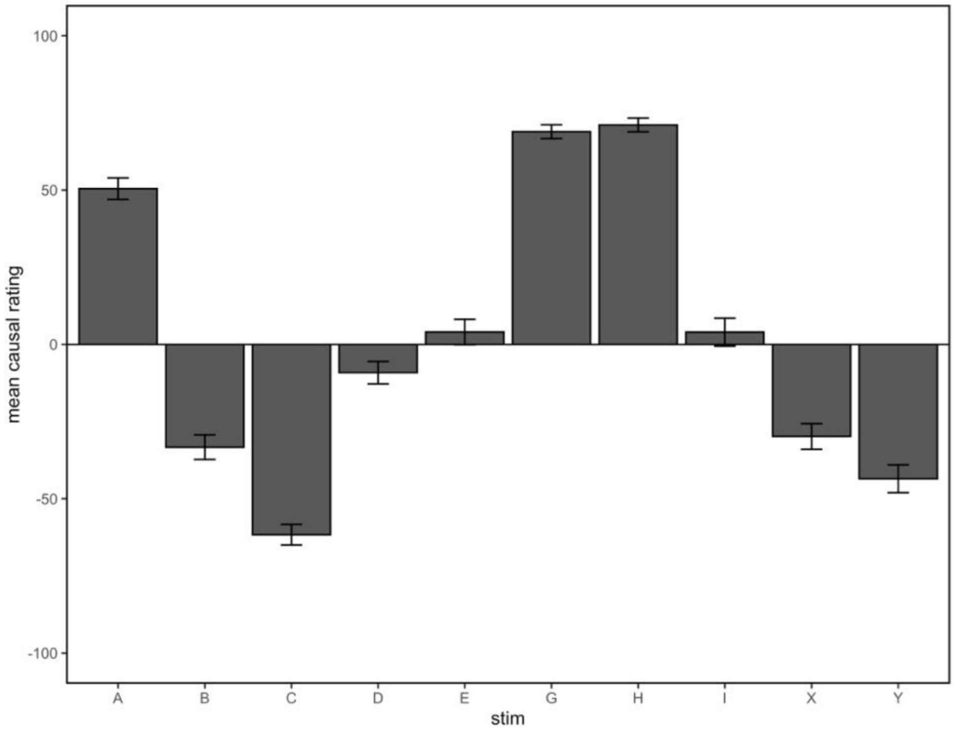

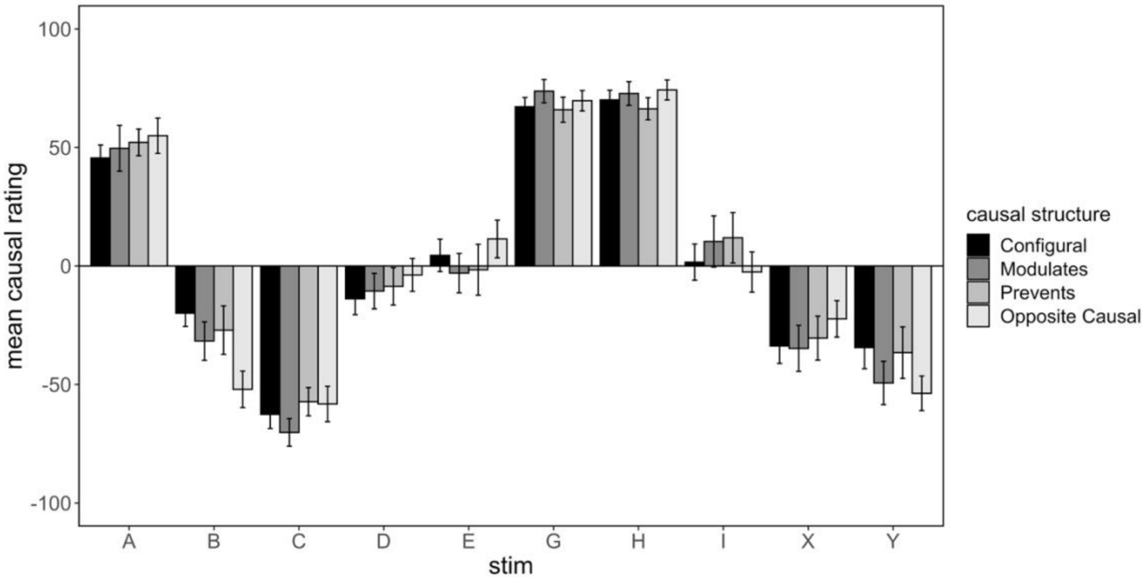

X vs Y

Opp vs Others

Main effect, $F(1,114) = .038$, $p = .847$, $\eta_p^2 < .001$

X vs Y interaction, $F(1,114) = 4.53$, $p = .035$, $\eta_p^2 = .038$

Config vs Inhib

Main effect, $F(1,114) = .194$, $p = .660$, $\eta_p^2 = .002$

X vs Y interaction, $F(1,114) = .596$, $p = .441$, $\eta_p^2 = .005$

B vs D

Opposite vs Others

Main effect, $F(1,114) = 2.41$, $p = .124$, $\eta_p^2 = .021$

B vs D interaction, $F(1,114) =8.72$, $p = .004$, $\eta_p^2 = .072$

Configural vs Inhibitory

Main effect, $F(1,114) = .157$, $p = .693$, $\eta_p^2 = .001$

B vs D interaction, $F(1,114) = 1.24$, $p = .268$, $\eta_p^2 = .011$

Cue B alone

Opposite vs Others $F(1,114) = 9.38$, $p = .003$, $\eta_p^2 = .076$
Configural vs Inhib, $F(1,114) = 1.04$, $p = .309$, $\eta_p^2 = .009$
